# Aire-dependent interferon signalling shapes thymocyte maturation and central tolerance in mice

**DOI:** 10.1038/s42003-025-09317-9

**Published:** 2025-12-09

**Authors:** Adrianna Jebrzycka, Lars Breivik, David Dolan, Yael Goldfarb, Jakub Abramson, Anette S. B. Wolff, Eystein S. Husebye, Anagha Joshi, Bergithe E. Oftedal

**Affiliations:** 1 Department of Clinical Science, University of Bergen, Bergen, Norway; 2 Department of Medicine, Haukeland University Hospital, Bergen, Norway; 3 Department of Informatics, Computational Biology Unit, University of Bergen, Bergen, Norway; 4 Department of Immunology and Regenerative Biology, Weizmann Institute of Science, Rehovot, Israel; 5 Health Research Sogn og Fjordane, Førde Hospital Trust, Førde, Norway

**Keywords:** Immunology, Genetics

## Abstract

Autoimmune regulator (Aire) orchestrates the presentation of self-antigens to developing thymocytes, playing a key role in central tolerance. However, it remains unclear how Aire deficiency influences immune cell development and function. Recent studies show that Aire-expressing thymic epithelial cells produce type I and III interferons, but how impaired IFN signalling from Aire deficiency contributes to immune dysregulation remains unclear. Single-cell RNA sequencing was used to profile immune cells from Aire-deficient and wild-type mice across thymus, bone marrow, and lymph nodes. In the thymus, thymocytes at late maturation stages, non-conventional T cells, myeloid cells, immature Ccl21+ mTECs and cTECs had reduced expression of type I interferon-stimulated genes in the absence of Aire. In contrast, interferon-stimulated gene expression in bone marrow immune cells appeared independent of Aire. These findings support a role for Aire in thymic interferon production and highlight type I IFNs’ influence on transcriptomes of developing immune cells.

## Abstract (web summary)

Single cell RNA sequencing reveals reduced expression of type I interferon-stimulated genes in late stage thymocytes and thymic epithelial cells when Aire is deficient, suggesting a role for Aire in thymic interferon production.

## Introduction

A key regulator of central T cell tolerance is the autoimmune regulator (Aire), a transcriptional activator mainly expressed in medullary thymic epithelial cells (mTECs). Aire promotes the expression of tissue-restricted antigens (TRAs), enabling the thymus to present a broad array of self-antigens to developing thymocytes^1^. Loss of Aire function leads to the escape of autoreactive clones and the development of multiorgan autoimmunity in both mice and humans^2–6^. Beyond its role in promoting TRA expression, Aire has also been shown to influence the thymic cytokine milieu, including the expression of interferons (IFNs) and chemokines that shape immune cell positioning and function^4,7–12^.

T cell development in the thymus is a tightly regulated process in which thymocytes acquire diverse TCRs and functional competence. As they progress through the double-negative (DN), double-positive (DP) and single-positive (SP) stages, thymocytes undergo selection checkpoints that test TCR specificity for both functionality and self-tolerance^13^. Positive selection in the cortex ensures survival of thymocytes that recognise self-MHC, while negative selection in the medulla eliminates or redirects clones with high affinity for self-antigens. These processes are supported by a specialised thymic microenvironment composed of cortical and medullary thymic epithelial cells (cTECs and mTECs) and hematopoietic antigen-presenting cells (APCs)^14,15^.

Recent findings suggest that Aire regulates the constitutive production of type I and III IFNs by mTECs, which are thought to influence thymocyte maturation, antigen presentation and Treg selection, as supported by observations in IFN receptor-deficient mice, indicating their important contribution to the establishment of central T cell tolerance^9,10,12^. Nevertheless, the extent to which IFN signalling is disrupted in the Aire-deficient thymus and its impact on thymocyte development, as well as APCs within the hematopoietic and stromal compartments in this model, remains poorly understood. Previous studies have suggested defects in thymic APC accumulation, regulatory T cell induction, and thymocyte maturation in Aire-deficient mice^8,11,16–21^, but a detailed, high-resolution understanding of the thymic immune cell landscape upon loss of Aire is still largely lacking. Additionally, low levels of *Aire* expression have been detected in cells within peripheral lymphoid organs, including lymph nodes (LNs) and spleen^1,6,22^. Yet, the exact phenotype of these extrathymic Aire-expressing cells or their potential in maintaining tolerance or shaping immune cell composition and function in these organs remains unclear, with existing data being both conflicting and limited^22–25^.

In this study, we aimed to dissect the immune cell composition and transcriptional programs of Aire-deficient lymphoid organs at single-cell resolution. We uncover a stage-specific reduction in IFN signalling in developing thymocytes, thymic myeloid cells and in subpopulations of TECs, highlighting a previously underappreciated role for Aire in sustaining a thymic IFN niche. Interestingly, this defect appears to be thymus-specific, as the IFN signature in bone marrow (BM) immune cells appears unaffected by Aire expression, and the loss of IFN signalling in the thymus does not further imprint the T cells in the periphery of young mice. These findings suggest that IFN signalling plays a crucial role in how Aire influences thymocyte development and central tolerance.

## Results

### Single-cell profiling of murine thymic immune cells depicts developing thymocytes and minor APC populations

**Fig. 1:**
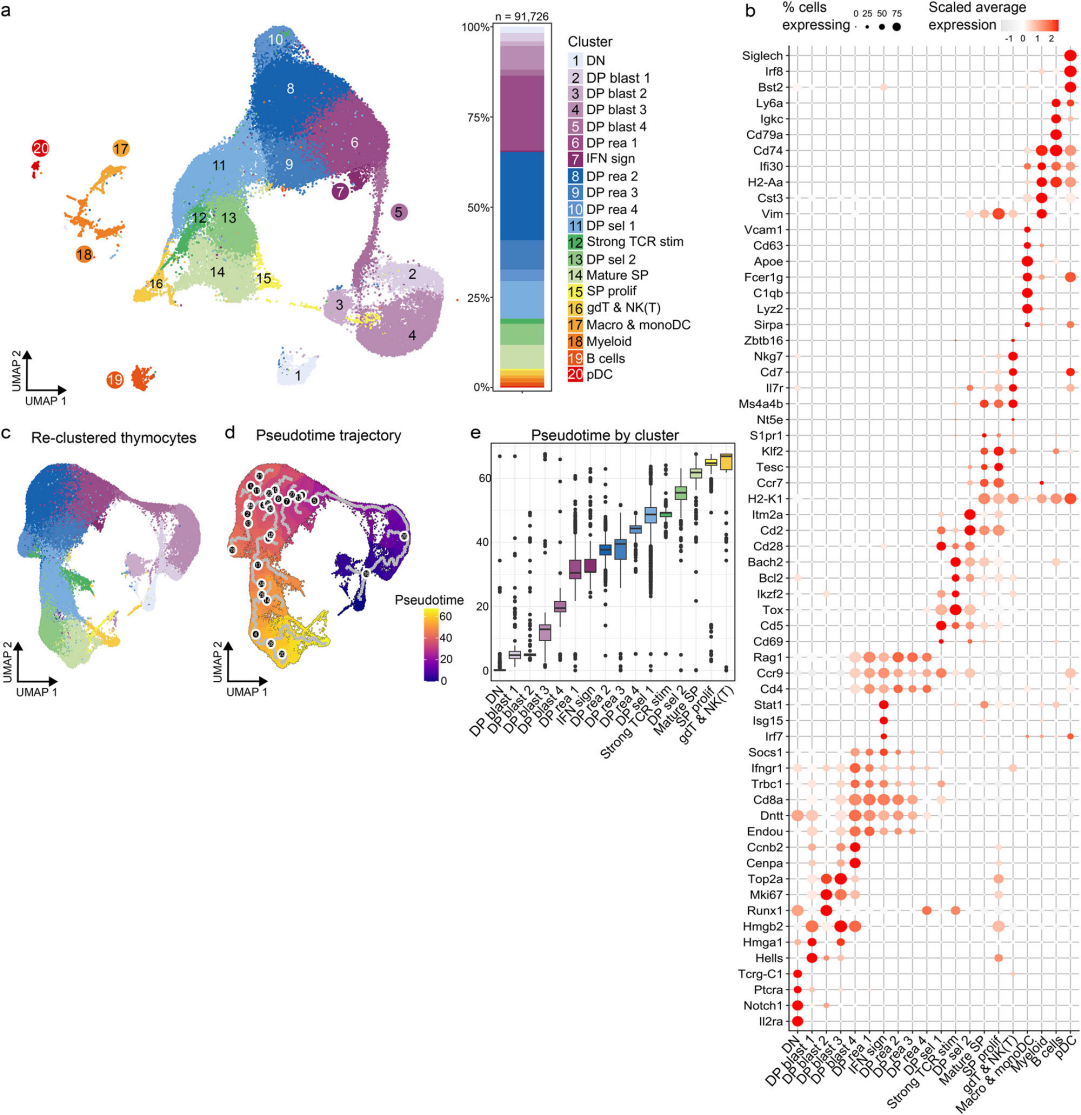
Single-cell profiling of thymic immune cells from Aire-deficient (Aire^C313X−/−^) and wild-type (WT) mice. **a** Low-dimensional (UMAP) representation of thymic immune cell populations in WT and Aire^C313X−/−^ mice, depicted in a shared UMAP space and their relative abundance per cluster. **b** Dotplot of conserved marker genes for the 20 thymic clusters. Size of dot represents the percentage of cells in clusters expressing a gene, while the colour represents the scaled average expression level. **c** UMAP plot of thymocytes after re-clustering, retaining cluster labels from the main thymus object. **d** Developmental trajectory graph calculated using Monocle3, coloured by pseudotime. **e** Boxplot depicting ordering of thymocyte clusters, based on increasing median pseudotime values.

To dissect how Aire impacts the composition and transcriptomes of thymic immune cells, we single-cell sequenced ~10,000 thymic immune cells in four Aire-deficient and four WT mice. Twenty distinct populations were identified, including 16 thymocyte clusters representing different developmental stages and four minor hematopoietic APC clusters (Fig. 1a, b). Early thymocytes included DN and proliferating DP blast cells, while DP cells undergoing TCR rearrangement formed the largest proportion of the dataset (Supplementary Fig. 1a–d). An increase in expression of *Rag1* (Supplementary Fig. 1e) marked the transition to DP cells undergoing TCR rearrangements, clusters termed DP rearranging (DPrea). Later stages comprised thymocytes undergoing selection, mature SP cells, and a common cluster of gamma delta (gdT) and NK(T) cells (Fig. 1b and Supplementary Fig. 1f–h). APC populations included B cells, macrophage/monocytic DCs, plasmacytoid DCs and myeloid-activated cells.

Developmental progression was predicted using pseudotime trajectory analysis using Monocle3^26^. Sixteen thymocyte clusters were subsetted and re-clustered (Fig. 1c). The developmental trajectory starting from the DN cluster expressing *Il2ra* and *Notch1* (Supplementary Fig. 1i, j) revealed the expected transitions from early proliferation to TCR rearrangement and selection (Fig. 1d, e and Supplementary Fig. 1k, l). Transcriptomic and TCR-seq data showed a gradual increase in paired-chain TCR expression, marking thymocyte maturation (Supplementary Fig. 1m). However, only around 50% of clones completing TCR rearrangement expressed paired-chain receptors.

### Aire deficiency affects gene expression profiles of thymocytes in final maturation stages and thymic APCs

**Fig. 2:**
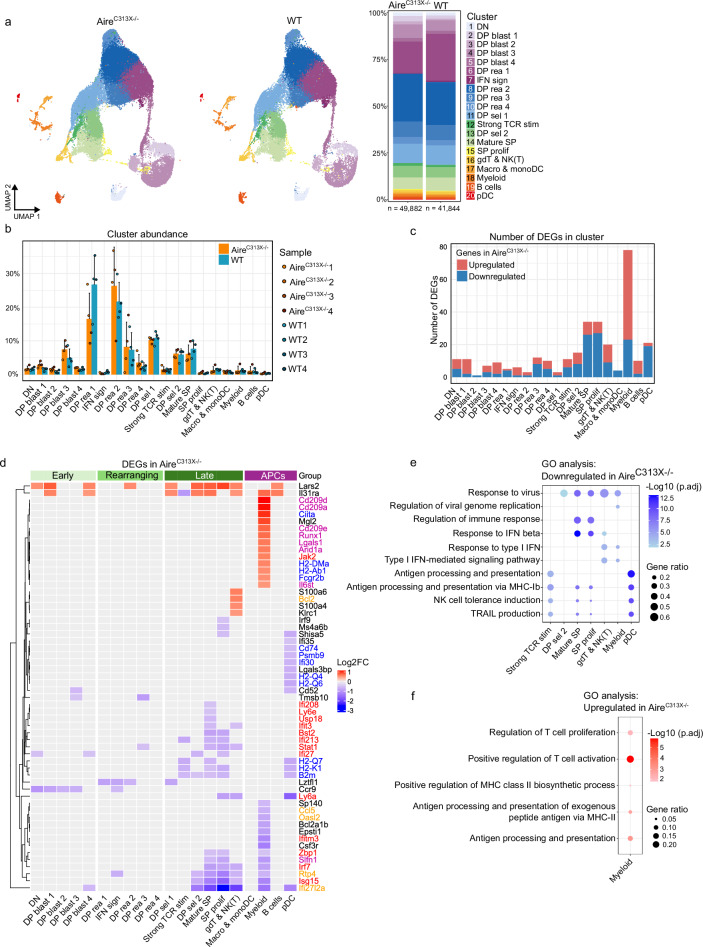
Comparison of thymic immune cell profiles across wild-type (WT) and Aire-deficient (Aire^C313X−/−^) mice. **a** UMAP plots of immune cell populations in Aire^C313X−/−^ and WT mice. **b** Proportion of cells in clusters compared across Aire^C313X−/−^ and WT (*n* = 4 per genotype, Wilcoxon rank sum test with Benjamini–Hochberg correction, ns). Error bars in bar plots indicate standard deviation. **c** Number of significantly differentially expressed genes (DEGs) per cluster, red marks upregulated and blue downregulated DEGs in Aire^C313X−/−^. **d** Heatmap displaying log2fc values for selected DEGs across Aire^C313X−/−^ and WT, for full overview see Supplementary Data 1.3. Colour scale red: upregulated and blue: downregulated genes in Aire^C313X−/−^. Gene names coloured by corresponding GO terms from Fig. 2e, f: red: response to type I IFN/IFN-beta, yellow: response to virus, blue: antigen processing and presentation, purple: regulation of T cell activation. Top GO terms enriched among **e** downregulated genes and **f** upregulated genes for clusters with more than 10 DEGs total. As many of the GO terms showed considerable degree of redundancy, we only show terms we deemed most relevant, for full overview see Supplementary Data 1.4 and 1.5). GeneRatio indicates the ratio of genes in term among DEGs, while log10 for adjusted *p* values are indicated by the colour scale (over-representation test, a version of Fisher's exact test with Benjamini–Hochberg correction).

To determine whether Aire deficiency impacts thymic immune cell composition, the relative cell abundance across clusters was calculated for each individual mouse and compared between Aire^C313X−/−^ and WT mice (Fig. 2a). The abundance of cells per cluster was similar in both groups, also the selecting thymocyte (DPsel1 and DPsel2) and mature SP clusters were similar, indicating that defects in negative selection do not influence cell numbers during or after selection (Fig. 2b and Supplementary data 1.1). TCR sequencing confirmed similar repertoire diversity across genotypes, with no biases in diversity or V and J gene usage (Supplementary Fig. 2 and Supplementary Data 1.2). Taken together, analysis of thymic immune cell composition revealed comparable cluster abundances between Aire-deficient and WT mice, suggesting intact thymocyte development.

Differential gene expression analysis across clusters together identified 155 unique differentially expressed genes (DEGs), with few changes in early thymocyte stages but significant downregulation of genes in mature SP thymocytes and APCs (Fig. 2c, d and Supplementary Data 1.3). Key downregulated genes in Aire-deficient mice included interferon-stimulated genes (ISGs) and MHC-I molecules, affecting pathways related to IFN response and antigen presentation (Fig. 2e and Supplementary Data 1.4). Among APC clusters, plasmacytoid DCs showed reduced expression of antigen presentation-associated genes, while myeloid cells exhibited upregulation of T cell activation markers (Fig. 2d–f and Supplementary Data 1.5). Overall, Aire-deficiency leads to reduced IFN signature in late-stage thymocytes and APCs, possibly impacting antigen processing and presentation via MHC-I and MHC-II.

### Aire deficiency leads to reduced thymic interferon signature in late stage thymocytes

**Fig. 3:**
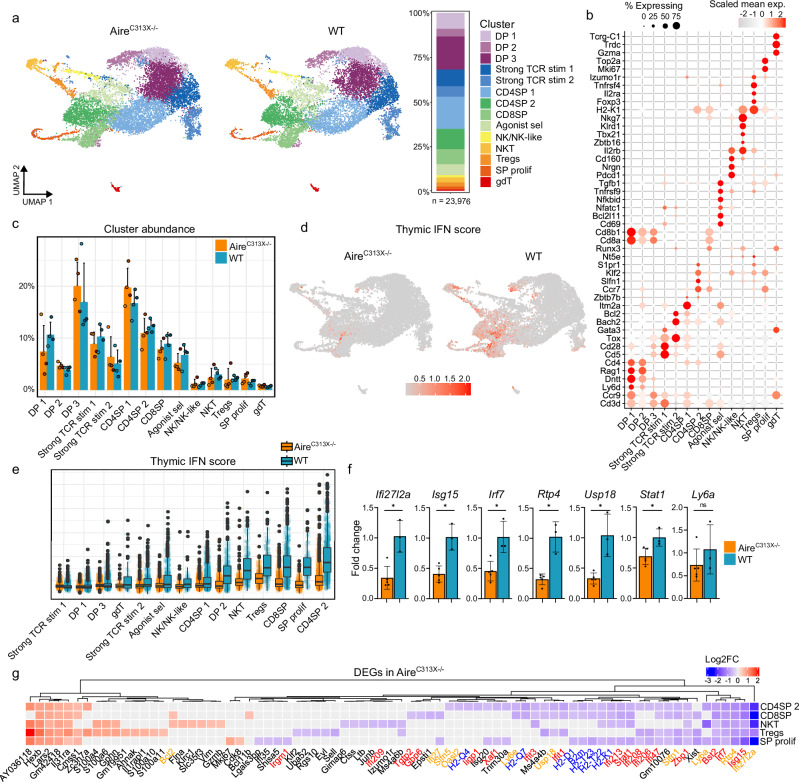
Reduction in interferon signature in re-clustered late-stage thymocytes from Aire-deficient (Aire^C313X−/−^) compared to wild-type (WT) mice. **a** Late-stage thymocytes consisting of cells annotated as DPsel 1, DPsel 2, Strong TCR stim, Mature SP, SP prolif and gdT/NK in the main thymus object (Fig. 1a) were subsetted and re-clustered yielding 14 higher-resolution clusters. **b** Dotplot of conserved marker genes for the 14 re-clustered late-stage thymocyte clusters. Size of dot represents the percentage of cells in cluster expressing a gene, while the colour represents the scaled average gene expression level. **c** Proportion of cells from each mouse for the 14 clusters, compared across Aire^C313X−/−^ and WT, *n* = 4 (Wilcoxon rank sum test with Benjamini–Hochberg correction, ns). **d** UMAP visualisation and **e** distribution of thymic interferon (IFN) module scores based on 15 most downregulated interferon-stimulated genes between Aire^C313X−/−^ and WT mice, clusters ordered by increasing median values for WT, number of cells are found in Supplementary Data 1.15. In UMAP common colour scale for Aire^C313X−/−^ and WT mice was used. **f** Expression (qPCR) of selected ISGs *Ifi27l2a*, *Isg15*, *Rtp4*, *Irf7*, *Usp18*, *Stat1*, *Ly6a* in bulk thymocytes from Aire^C313X−/−^ (*n* = 5) and WT (*n* = 3) mice (Wilcoxon rank sum test) etc. **g** Heatmap with log2fc values of the 77 differentially expressed genes (DEGs) in the five clusters with highest reduction in Interferon score. Colour scale red: upregulated and blue: downregulated genes in Aire^C313X−/−^. Gene name colours correspond to GO terms red: response to IFN beta/type I IFN, orange: response to virus, blue: MHC-I protein complex. Error bars in bar plots indicate standard deviation.

To further identify the subsets of late-stage thymocytes with the most severe reduction in IFN signalling, 22,976 late-stage thymocytes were subsetted and re-clustered, yielding 14 refined clusters (Fig. 3a). The clusters were annotated by markers specific for localisation within the thymus (*Ccr9*, *Ccr7*), selection-induced TCR signalling (*Cd5*), maturation (*H2-K1, Cd69*), egress (*Klf2, S1pr1, Nt5e* (encoding CD73)) and lineage regulation (*Cd4, Cd8a, Foxp3, Zbtb16*) (Fig. 3b and Supplementary Fig. 3). Cell abundances in these higher resolution clusters were similar between WT and Aire^C313X−/−^ mice (Fig. 3c and Supplementary Data 1.6).

A thymic IFN module score based on the 15 most downregulated ISGs in late stage thymocytes (Supplementary data 1.7) termed ‘thymocyte ISG DEGs-based interferon score’ showed most pronounced loss of IFN stimuli in SP CD4 and CD8 thymocytes, proliferating SP cells, Tregs and NKT cells of Aire-deficient mice (Fig. 3d, e and Supplementary Data 1.8). Differential gene expression analysis of the higher resolution clusters together with qPCR analysis of bulk thymocytes confirmed downregulation of key ISGs, including *Ifi27l2a*, *Isg15*, *Rtp4*, *Irf7*, *Usp18* and *Stat1*, alongside reductions in antigen presentation pathways (Fig. 3f, g and Supplementary Data 1.9–1.11). Tregs also showed lower expression of egress-related genes (*Klf2*, *Cd62l*), while CD8SP and NKT cells exhibited increased anti-apoptotic (*Bcl2*) and effector (*Gzmb*, *Mki67*) gene expression (Fig. 3g). Overall, the diminished IFN signature in late-stage thymocytes and APCs might be linked to altered gene expression patterns affecting survival, proliferation, and immune function.

### Reduction in IFN signature is mirrored in Aire-deficient TECs

**Fig. 4:**
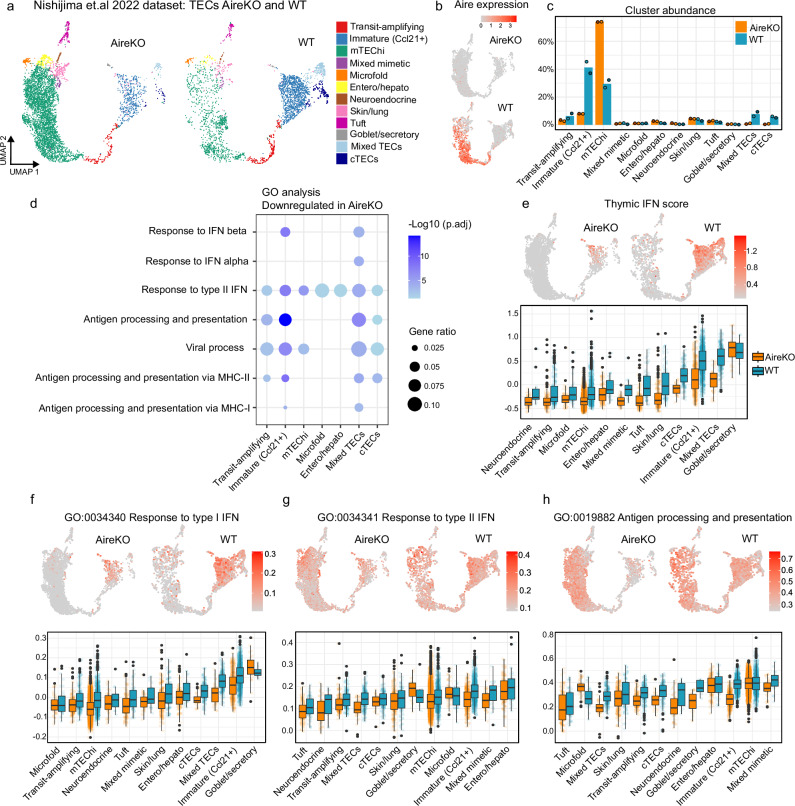
Reduction in interferon signature in thymic epithelial cells (TECs) from Aire-deficient (AireKO) compared to wild type (WT) mice. **a** UMAP plots of 12 clusters from publicly available TEC datasets from AireKO (*n* = 2) and WT (*n* = 2) mice (GSE155331). **b** UMAP plots displaying Aire expression. **c** Relative abundance of cells in clusters from AireKO and WT mice **d** GO terms enriched among downregulated genes in AireKO mice. GeneRatio indicates the ratio of genes in term among DEGs, while log10 for adjusted *p* values are indicated by the colour scale (over-representation test, a version of Fisher's exact test with Benjamini–Hochberg correction). UMAP visualisation (upper panels) and distribution of module scores (lower panels) based on **e** thymocyte ISG DEGs and genes within GO terms **f** ‘response to type I interferon’, **g** ‘response to type II interferon’ and **h** ‘antigen processing and presentation’. For UMAP plots in (**b**), **e**–**h** expression of features in AireKO and WT mice are shown on a common colour scale. For boxplots in **e**–**h**, clusters are ordered by increasing median values for WT, and *n* is found in supplementary data 2.10.

To determine whether the reduction in ISG expression also affects thymic epithelial cells, a dataset of sorted TECs from Aire knockout (AireKO) and WT mice was re-analysed^27^ (GSE155331), identifying twelve clusters^28^. These included transit-amplifying cells, immature mTECs (mTEC^lo^ cells, expressing high levels of *Ccl21a*), mTEChi, cTECs, mixed TECs (expressing *Ackr4*, *Prelp*, *Cxcl12*, *Il33* and *Pdpn*) and several mimetic cell populations (Fig. 4a, Supplementary Fig. 4 and Supplementary Data 2.1). The composition of TEC populations in AireKO and WT mice differed greatly, with a higher abundance of mTEChi (but Aire negative, Fig. 4b) and a decrease in immature mTECs, cTECs and mixed TECs in AireKO mice, as commonly seen for Aire deficient mice strains^20,29,30^ (Fig. 4c and supplementary Data 2.2).

Gene Ontology (GO) analysis confirmed downregulation of viral and IFN response pathways among TECs, now including genes regulated by both type I and type II IFNs (Fig. 4d; Supplementary Data 2.3 and 2.5). We employed the thymocyte ISG DEGs-based IFN score on the TEC dataset (Fig. 4e) and observed a significant reduction in IFN score in almost all TEC populations, the greatest in mixed TECs, immature Ccl21+ mTECs, Tuft, Skin/lung mimetics and cTECs (Supplementary Data 2.6). To further dissect the expression pattern of ISGs regulated by type I and type II IFNs two new IFN response module scores based on genes within their respective GO terms (Supplementary Data 2.7 and 2.8). While the type II IFN response module showed ubiquitous expression of the associated ISGs within most TEC populations, the type I IFN response score was mostly restricted to Immature mTECs, cTECs and mixed TECs (Fig. 4f, g), notably overlapping with the thymic IFN score based on ISG DEGs in thymocytes. Additionally, Immature mTECs, cTECs and mixed TECs, were much less abundant in AireKO than WT mice (Fig. 4c). Reduction of IFN responses correlated with downregulation of antigen processing and presentation pathways, where the reduction was most pronounced in type I IFN responding TEC populations as determined by module score based on genes constituting the ‘antigen processing and presentation’ GO term (Fig. 4h and Supplementary Data 2.9). Overall, these data suggest a critical role of thymic IFNs in shaping the TEC transcriptomes and their antigen-presenting capabilities.

### Constitutive interferon signalling in immune cell progenitors in the bone marrow is independent of Aire

**Fig. 5:**
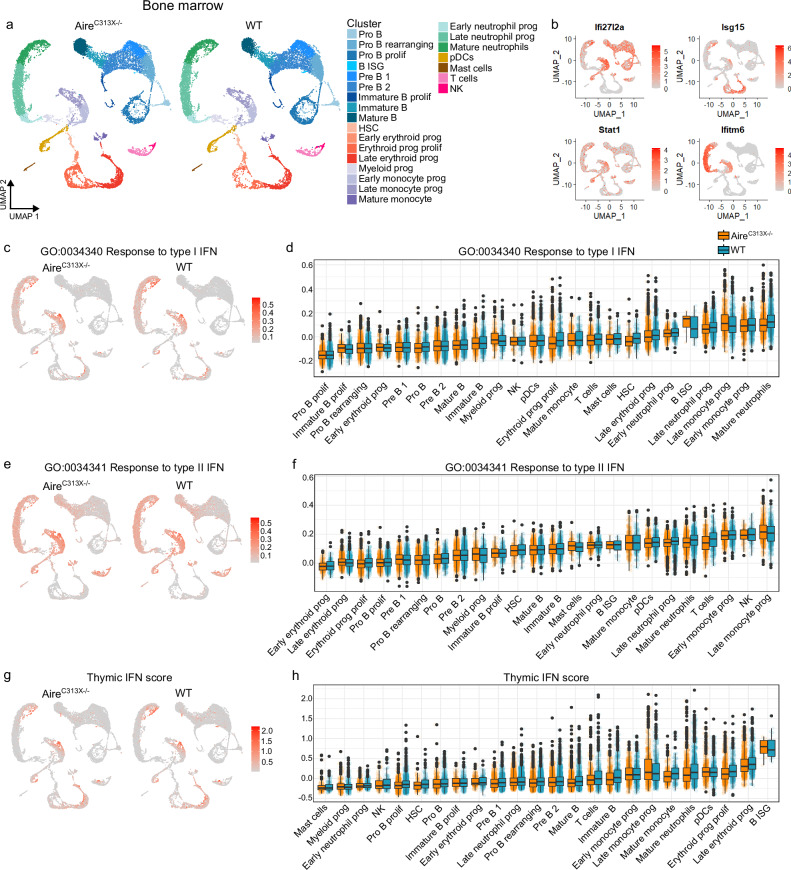
Immune cells isolated from the bone marrows (BM) of Aire^C313X−/−^ and wild type (WT) mice similarly respond to interferons at steady state. **a** UMAP plots of BM immune cells in Aire^C313X−/−^ and WT mice. **b** Normalised expression of exemplary interferon-stimulated genes (ISGs) *Ifi27l2a*, *Isg15*, *Stat1*, *Ifitm6* in shared UMAP space of WT and Aire^C313X−/−^ BM immune cells. UMAP visualisation and distribution of interferon (IFN) module scores in BM immune cell populations based on genes within GO terms **c**, **d** ‘response to type I interferon’, **e**, **f** ‘response to type II interferon’, or thymocyte ISG DEGs in (**g**, **h**) thymic IFN score. For UMAP plots in (**b**, **d**, **f**, **h**), expression of the feature in Aire^C313X−/−^ and WT mice are shown on a common colour scale. For boxplots in (**d**, **f**, **h**), clusters are ordered by increasing median values for WT, and *n* are found in Supplementary Data 3.10.

To investigate whether IFN production is common for the primary lymphoid organs, BM from the same Aire-deficient and WT mice were analysed. Single-cell RNA sequencing dataset encompassing 32,086 BM immune cells identified 25 distinct clusters, including hematopoietic stem cells (HSCs), developing B cells, erythroid cells, monocytes, neutrophils, and various mature immune subsets (Fig. 5a, Supplementary Fig. 5a, b and Supplementary Data 3.1). Several progenitor populations showed high basal ISG expression, especially neutrophil and monocyte progenitors (Fig. 5b). No dysregulation in pathways relating to IFN or viral response was found, suggesting that IFN production in the BM is intact in Aire-deficient mice, as supported by qPCR measurements of ISGs (supplementary Fig. 5c; Supplementary Data 3.2 and 3.5). The type I and type II IFN module scores were comparable across the BM clusters (Fig. 5c–f; Supplementary Data 3.6 and 3.7). Lastly, the thymocyte ISG DEGs-based IFN score was used to determine whether the DEGs in late-stage thymocytes were already altered prior to thymic entry of T cell progenitors, but no major differences in BM progenitor populations across WT and Aire-deficient mice were identified (Fig. 5g, h and Supplementary Data 3.8). These findings indicate that immature immune progenitors are exposed to tonic IFNs in the BM independently of Aire and support that the reduced thymic IFN signature observed in Aire-deficient mice is generated within the thymus itself.

### Reduction in IFN signature in Aire^C313X−/−^ mice is thymus specific

**Fig. 6:**
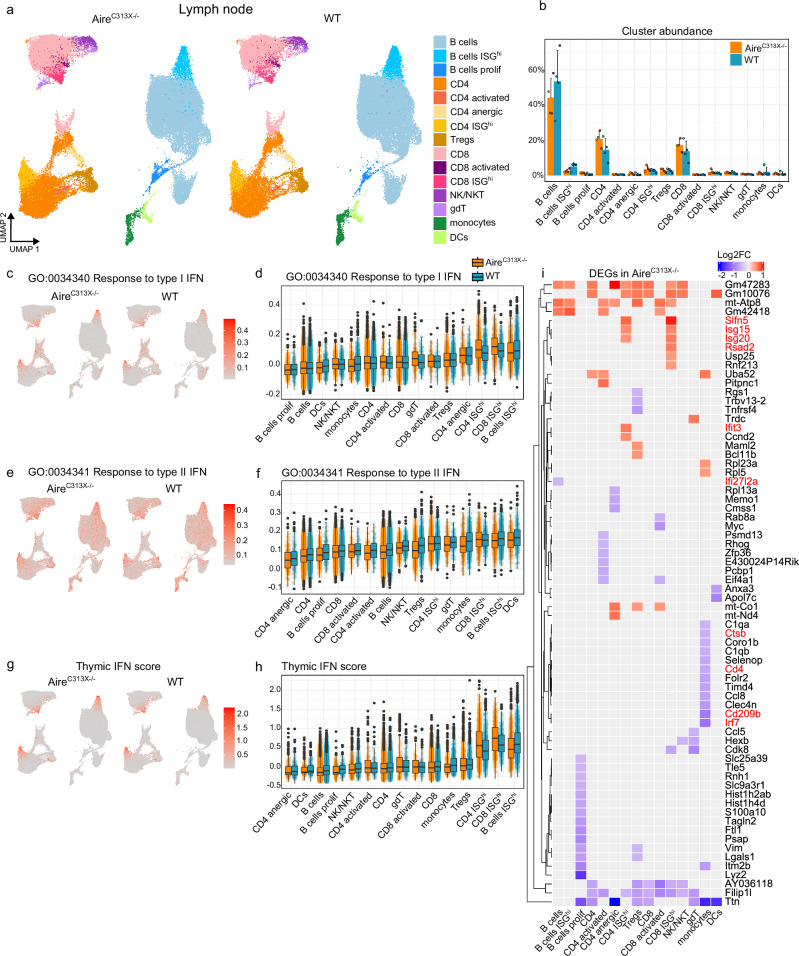
Immune cells isolated from the lymph node (LN) of Aire^C313X−/−^ and WT mice display similar interferon signature across genotypes. **a** UMAP plots of LN immune cells in Aire^C313X−/−^and WT mice. **b** Relative abundance of cells in LN clusters across Aire^C313X−/−^ UMAP and WT mice, *n* = 4 per genotype. UMAP visualisation and distribution of interferon (IFN) module scores in LN immune cells in Aire^C313X−/−^ and WT mice for **c**, **d** ‘response to type I interferon’, **e**, **f** ‘response to type II interferon’, or **g**, **h** thymocyte ISG DEGs. **i** Heatmap with log2fc values of differentially expressed genes (DEGs) in the LN dataset. Colour scale red: upregulated and blue: downregulated genes in Aire^C313X−/−^ mice. Gene names coloured red are DEGs corresponding to enriched GO terms ‘response to virus’. For UMAP plots in (**c**, **e**, **g**), expression of features in Aire^C313X−/−^ and WT mice are shown on a common colour scale. For boxplots in (**c**, **e**, **g**), clusters are ordered by increasing median values for WT, for *n* please see Supplementary Data 4.9.

To assess whether the reduced thymic IFN stimulation affects ISG expression in mature T cells following thymic egress, we analysed scRNA-seq profiles of 89,751 LN immune cells from the same Aire^C313X−/−^ and WT mice. Fifteen clusters were identified, mainly B cells, CD4 + T cells, and CD8 + T cells (Fig. 6a and Supplementary Fig. 6a). Three small lymphocyte populations were characterised by high expression of ISGs as cluster marker genes, namely B cells ISG^hi^, CD4 ISG^hi^ and CD8 ISG^hi^. ISG^hi^ CD4 and CD8 T cells showed a mild, non-significant increase in frequency of total LN cells in Aire-deficient mice, whereas ISG^hi^ B cells exhibited the opposite trend (Fig. 6b).

The IFN type I and type II module scores remained largely similar across genotypes (Fig. 6c–f), with subtle increase for the type I IFN GO module and thymic IFN signature within CD4 ISG^hi^ and CD8 ISG^hi^ clusters in Aire^C313X−/−^ mice (Supplementary Data 4.1 and 4.3). Despite a few upregulated ISGs in ISG^hi^ T cells (Fig. 6i and Supplementary Data 4.4), no functional impact was identified by the GO pathways analysis (Supplementary Data 4.5 and 4.6). qPCR of bulk LN immune cells did not confirm ISG expression differences between Aire-deficient and WT mice (Supplementary Fig. 6b and Supplementary Data 4.7). This indicates that the responses to IFNs produced in the LN environment/periphery are similar in Aire-deficient and WT mice.

Taken together, these findings suggest that IFNs are produced in primary lymphoid organs, and a reduced IFN signature is evident when Aire expression is absent in the thymus. Additionally, thymic IFN stimulation appears to have minimal impact on peripheral T cell transcriptomes in young Aire-deficient mice.

## Discussion

This single-cell transcriptomic analysis of immune cells from young Aire-deficient mice reveals that the effects of Aire loss are confined to the thymus, with minimal impact on immune progenitors in the BM and peripheral LN immune cells. Early thymocyte development and gene expression profiles before selection remain intact, indicating no phenotypical or functional defects. However, at late maturation stages, SP CD4 and CD8 thymocytes, Tregs and NKT cells exhibit a notable reduction in ISG expression. Similarly, Aire-deficient TECs and myeloid cells show diminished IFN signatures, while other thymic hematopoietic APCs remain unaffected. Although some ISGs were upregulated in peripheral LN T cells, the absence of relevant enriched pathways suggests that thymic IFN stimulation has little effect on the transcriptomes of mature T cells in Aire-deficient mice.

During thymocyte development in the thymus, our main finding is the consistent downregulation of type I IFN and viral response genes in Aire-deficient medullary thymocyte populations. This supports the proposed role of type I IFNs in late-stage maturation of CD4 thymocytes as suggested by Xing et al.^31^, which we here extend to SP CD8, Treg and NKT cells. This further aligns with the described diminished expression of type I and type III IFNs in Aire+ mTEC^hi^ cells in mice^9^.

Aire-deficient thymocytes have reduced expression of genes encoding classical and non-classical MHC-I. Previous studies linked lower MHC-I and Qa2 expression to defects in late-stage thymocyte maturation, however, our transcriptomic data only show minor, non-significant changes in mature SP thymocyte abundance^17–21^. We have previously published flow cytometry data showing reduced MHC-I expression in late maturational stages in thymocytes from Aire^C313X−/−^ mice^20^. The reduced MHC-I expression in Aire-deficient mice may result from diminished Aire-regulated IFN production in the thymic medulla, aligning with known roles of IFNs and Stat1 in promoting MHC-I expression in thymocytes^32^. Thymic egress appears largely unaffected in adult Aire-deficient mice, as egress markers such as *Nt5e*, *Klf2* and *S1pr1* remain unchanged^33^, except for reduced *Klf2* and *Sell* in Tregs. Together, our findings largely align with previous reported data^21^, as we do not see clear disturbances in the expression of egress-related markers, indicating thymic egress is likely not disturbed.

By re-analysing a single-cell dataset of TECs from 8-week-old AireKO and WT mice, we found that the reduction of thymic IFN responses is reflected in the TEC compartment. Contrary to late-stage thymocytes, GO analysis indicated both type I IFN and type II IFN responses to be diminished in TECs. This might suggest reduced thymic IFN gamma production in the TEC compartment of Aire-deficient mice and warrants further investigation. Looking at the ISGs induced by either type I and type II IFNs showed that most TEC populations responded to type II IFNs, while type I responders were primarily restricted to immature Ccl21+ mTECs, mixed TECs and cTECs. Type I responding TECs were also less abundant in AireKO mice than in WT mice, suggesting that the reduction of type I IFNs might have a more pronounced effect on TEC transcriptomes within the Aire-deficient thymus than the reduction of type II IFNs. Intriguingly, a recent study on pathways directing TEC differentiation in the human thymus revealed that both type I and type II IFNs are central in this process^34^.

Besides thymocytes and TECs, myeloid cells were the only other thymic immune cell population where a reduction in IFN signature was evident in Aire-deficient mice. Ashby et al. recently showed that most hematopoietic APC populations in the thymus are highly responsive to constitutively produced IFNs, and that reduced IFN signalling in Ifnar^−/−^Ifnlr^−/−^ mice affects maturation of thymic macrophages, licensing of thymic B cells, and results in skewing of the cDC1/cDC2 ratio towards the latter^9,35^. Hints of increased number and/or functionality of cDC2s can also be seen in our dataset, as MHC-II molecules, the MHC-II transactivator *Ciita*, and *Mgl2* (encoding the cDC2 surface marker CD301b) are all upregulated in the myeloid cluster. Future studies with higher numbers of thymic hematopoietic APCs are needed to accurately represent their subpopulations and verify to what degree the reduction of type I and type III IFNs is reflected in their numbers and function in Aire-deficient mice.

Similar to the thymus in WT mice, we found high basal expression of ISGs among developing immune cell populations in the BM, indicating that both primary lymphoid organs harbour a pro-inflammatory environment at steady state. Contrary to the thymus, the ISG signatures in the BM were not affected in Aire-deficient mice, indicating that the reduced thymic IFN signature is not a result of IFN exposure at the T cell progenitor stage, prior to thymic entry. Following the T cells after egress to the periphery, ISG^hi^ profiles could be observed among B cells, CD4^+^, and CD8^+^ T cells in the LN, but were also largely Aire-independent. Future studies tracking recent thymic emigrants could clarify the impact of thymic IFN stimulation on naïve T cell fate and IFN responsiveness in the periphery.

The steady-state thymic production of pro-inflammatory cytokines and Aire’s possible role in this is intriguing. Two recent studies suggest this pro-inflammatory environment may help thymocytes develop tolerance towards the self-proteome transiently expressed during infections^9,36^. Although the detection of type I IFNs directly is difficult due to their low expression levels, IFN-α has been described at the protein level in thymic pDCs, found to be significantly decreased in Aire-deficient mice^37^. Whether the pDC, mTECs, or other APCs are the main contributors to IFN-α expression remain to be established. Interestingly, a down-regulation of ISGs in mTECs of young Aire-deficient rats was recently described, and like our data, this reduction was not recapitulated in LNs^38^. Further, these rats developed autoantibodies against IFN-α upon increasing age, appearing after the reduced thymic IFN signature^38^, while these autoantibodies are absent in pathogen-free housed mice^39^. Humans with pathogenic *AIRE* mutations similarly develop autoantibodies against cytokines, including type I IFNs^40–42^, suggesting that Aire has a role in tolerance development towards infection-induced self-antigens.

In summary, thymocyte development remains largely intact in Aire-deficient mice, with hematopoietic APC populations being similar to WT mice. However, Aire deficiency alters thymic immune cell transcriptomes, leading to reduced IFN-stimulated gene expression in SP CD4 and CD8 cells, Tregs, NKT cells and myeloid cells. This diminished IFN signature due to the lack of Aire expression is also evident in subpopulations of TECs but not in BM or LN immune cells, indicating its restriction to the thymus. Our findings support the role of Aire in thymic IFN production and highlight type I IFNs’ influence on thymic immune cell transcriptomes.

Although gene expression profiles provided sufficient information for the developmental ordering of thymocytes in our study, additional information on the protein level of marker genes would have enhanced our ability to more accurately define their phenotype for comparisons with previously reported flow cytometric studies. Enrichment of hematopoietic APCs would be needed to capture all thymic APC populations and allow us to better discriminate populations in which conserved marker genes did not clearly point to one specific cell subset, and would have improved the power of statistical analyses. We also acknowledge that direct measurements of IFN themselves, rather than ISGs as a proxy for diminished IFN production in the Aire-deficient thymus, would have been preferred. Lastly, given the large theoretical diversity of clones that can arise during V(D)J recombination and the relatively low number of thymocyte clones with paired-chain TCR information in our data, we did not deem it appropriate to report on cluster-wise repertoire comparisons and therefore only compare ‘global’ TCR repertoire features in Aire^C313X−/−^ and WT mice.

## Methods

### Mouse models

Aire-deficient mice (B6.Aire^C313X−/−^) were generated in the transgene mouse facility at The Weizmann Institute of Science, using CRISPR/Cas9 genome editing in isolated 1-cell embryos from C57Bl/6 as described in ref. ^29^. It was previously established that the Aire^C313X−/−^ mice had no Aire expression and a significant decrease in expression of TRAs, the immune cell composition in the thymus and in the periphery was also analysed by flow cytometry^20,29^. All mice were bred and maintained at the animal facility of the University of Bergen under license from the Norwegian Food Safety Authority (13570) and were provided with standard rodent chow and autoclaved water ad libitum. We have complied with all relevant ethical regulations for animal use. All mice were sacrificed between 6 and 9 weeks of age, and all wild-type controls were sex-matched littermates of the Aire^C313X−/−^ mice. Euthanasia was performed using carbon dioxide gas, with mice euthanized by gradual exposure to CO_2_ in a sealed chamber until the cessation of breathing was confirmed, followed by verification of death by neck dislocation according to Norwegian legislation. Both genders were used, and no animals were excluded. In total, 18 mice were used, 8 mice were used for the single cell experiment, where thymic immune cells from four mice (two male and two female) per genotype and between three and five Aire^C313X−/−^ and 3 WT mice per genotype were used for qPCR analysis. The experiments were not blinded.

### Isolation of immune cells

Thymi and axillary LNs were squashed and filtered through 70 μm MACS SmartStrainers (Miltenyi Biotech, cat. 130-110-916) and washed with 0.5% foetal bovine serum (FBS; Gibco, cat. 10082147) in phosphate-buffered saline (PBS). BM was flushed from the cavity of dissected tibia with 0,5% FBS in PBS and filtered through 70 μm MACS SmartStrainers. Dead cells were removed with the Dead Cell removal kit (Miltenyi Biotech, cat. 130-090-101). Cells not used for single-cell RNA sequencing were cryopreserved at −150 °C in 10% Dimethyl Sulfoxide (Sigma Aldrich, cat. D2650) in FBS.

### Single cell RNA library preparation

Ten thousand cells (counted in duplicate) from each thymus were prepared with Chromium Next GEM Single Cell 5’ Kit v2 (10x Genomics, cat. PN-1000286) and run on the Chromium Controller (10x Genomics). Gene expression libraries were prepared using the same single cell kits, and V(D)J libraries were prepared with Chromium Single Cell Mouse TCR Amplification Kit (10x Genomics, cat. PN-1000254) and additional Library Construction Kits (10x Genomics, cat. PN-000352). Libraries were indexed with the Dual Index Plate TT Set A (10x Genomics, cat. PN1000215). All quality control of libraries was performed using Tapestation 4200 (Agilent Technologies) with High Sensitivity D5000 Screen tape assays (Agilent Technologies, cat. 5067-5592).

### Sequencing

Single cell libraries were sequenced on a NovaSeq6000 system (Illumina) by the Genomics Core Facility at the University of Bergen, using paired end sequencing, (read 1: 26 bp, read 2: 90 cycles, dual index: 10 bp). Libraries were quantified on a Novaseq SP-100 flow cell (Illumina, cat 20028313). Gene expression libraries were pooled 6:1 with V(D)J libraries and spiked with 1% phiX (Illumina, cat. FC-110-3001) for quality control. Sequencing was performed on a NovaSeq S4-200 flowcell (Illumna, cat. 20040719), aiming for a total of 36,000 reads per cell. Sequencing data are available in the ArrayExpress database (http://www.ebi.ac.uk/arrayexpress) under accession number E-MTAB-14774.

Raw sequencing data (BCL) were demultiplexed using the Cell Ranger v.7.0.1 (10x Genomics) mkfastq pipeline. The Cell Ranger v.7.0.1 multi pipeline was used for alignment, filtering, counting of gene expression libraries and sequence assembly and clonotype calling on V(D)J libraries with refdata-gex-mm10-2020-A as the genome reference and refdata-cellranger-vdj-GRCm38-alts-ensembl-7.0.0 as the V(D)J reference.

### Quality control, integration and clustering

Data analysis was performed using *Seurat* v. 4.4.0^43^ under R v. 4.3.1. To ensure good quality of the data, we filtered out empty droplets, doublets and low-quality cells by keeping cells satisfying the following criteria: 400–4000 expressed genes (nFeature), 500–12,000 counts (nCount) and <20% mitochondrial gene content (before qc: 100,202 total cells, after qc: 93,118 total cells). Genes expressed by fewer than 10 cells were filtered out. Following quality control of thymic samples, 91,826 cells were retained and the samples integrated to perform clustering in a shared UMAP space, and manually annotated using acknowledged marker genes and cell states previously reported^44–46^. For LN and BM, the *DoubletFinder* v.2.0.4^47^ was used to identify and filter out doublets, with expected doublet ratio set to 15% for LN and 6% for BM, as several clusters were defined by mutually exclusive immune cell markers. Additionally, clusters where more than 50% of cells were scored as doublets were filtered out (LN before qc: 120,083 cells, after qc: 89,751 cells, BM before qc: 52,611 cells, after qc: 32,086 cells). Seurat object containing all samples was subsequently split into two layers: WT and Aire^C313X−/−^, the data was normalised (LogNormalize), and the 2000 most variable features were calculated for each layer, using the vst method. Layers were integrated using Seurat’s canonical correlation analysis (CCA) with dims set to 1:20 for both anchoring and integration^48^. The data was then scaled with ScaleData. Dimensionality reduction was first performed using principal component analysis, then with uniform manifold approximation and projection (UMAP) using the first 25 PCs covering 90% of the variance in the data^49^. FindNeighbours was run with 1:25 dims, and final clustering with FindClusters was performed with res = 0.5 (thymus), res = 1 (LN) and res = 1.25 (BM). Cluster-specific markers were found using FindConservedMarkers. For the thymus, one cluster (cluster 11) with low median feature counts, expressing both thymocyte and APC markers, in which 60% of the total of 1392 cells came from one individual WT mouse, was excluded from further analysis. Cluster markers and cluster-wise cell numbers and proportions from each mouse are shown in Supplementary Fig. 7; Supplementary data 1.12 and 1.13 (thymus), Supplementary Fig. 8; Supplementary data 3.9 and 3.10 (BM) and Supplementary Fig. 9; Supplementary data 4.8 and 4.9 (LN).

### Analysis of a publicly available mTEChi dataset from Aire knockout and wild-type mice

A dataset of sorted CD45^−^EpCAM^+^ TECs from 8-week-old Aire knockout (AireKO, *n* = 2) and WT mice (*n* = 2) (GSE155331) was re-analysed using filtering parameters, normalisation, integration and scaling described in the original publication^27^. Dimensionality reduction (UMAP) and FindNeighbours was run on the first 42 PCs and clustering was performed with res = 2, yielding 26 clusters. Annotation was performed in a semi-supervised manner using signature gene sets for TECs, including mimetic cell types from Supplementary Table 2 in Michelson et al.^28^, keeping genes with log2fc > 2, adj.*p.*value < 0.01 and expressed in at least 10% of cells within the signature. The filtered gene sets were used as input for the AddModuleScore function in *Seurat*, which estimates the likelihood of genes within the provided gene set being co-expressed in a single cell. Signature scores were then overlayed on UMAP and manually assigned to clusters, merging clusters where the same signature yielded high scores, resulting in 12 final clusters. Population termed mTEChi here corresponds to label ‘Aire-stage’ in Michelson et al.^28^, while cluster termed mixed TECs expressing *Ackr4*, *Prelp*, *Cxcl12*, *Il33* and *Pdpn* did not match any previously described TEC signatures. See Supplementary Data 2.1 and 2.10 for cluster markers and cluster-wise cell numbers and proportions.

### Sub-clustering of thymocytes, late stage thymocytes and bone marrow B cells

For focused analyses of (1) thymocyte developmental trajectory, (2) late-stage thymocytes and (3) developing B cells in the BM, all steps of the analysis described above were repeated on subsetted objects, for (1) excluding APC clusters (Macro/monoDC, Myeloid, B cells and pDCs), (2) retaining only late-stage thymocyte and unconventional T cell clusters (DPsel1, Strong TCR stim, DPsel2, Mature SP, SP prolif, gdT/NKT) and (3) retaining B cell clusters (ProB, ProB rearraning, ProB prolif, B IFN stimulated genes (ISG), PreB1, PreB2, ImmatureB prolif, ImmatureB and MatureB). Features in the subsetted objects were re-normalised and re-scaled, and the most variable features were recalculated before reintegration of the WT and Aire^C313X−/−^ layers, so as to only reflect the differences between cells in the subsetted data. The number of PCs for dimensionality reduction in UMAP was adjusted to cover 90% of variance in the respective datasets; for (1) dim = 16, (2) dim = 25 and (3) dim = 25. Resolution for clustering was also evaluated, to yield clusters composing biologically meaningful populations, finally set to (1) res = 0.75, (2) res =  0.5 and (3) res = 0.5. Cluster-specific markers for late-stage thymocytes were found using FindConservedMarkers. Cluster markers and cluster-wise cell numbers and proportions from each mouse are shown in Supplementary Fig. 10; Supplementary Data 1.14 and 1.15.

### Pseudotime trajectory analysis

Pseudotime trajectory analysis on the subsetted (1) thymocyte object and (2) BM B cells were performed using *Monocle3* v.1.3.4. ‘Learn graph’ function was run with partition = F and the pseudotime values were determined with ‘order cells’ setting (1) DN cells or (2) ProB cells as the root node. Final developmental order of thymocyte clusters and BM B cells was determined based on increasing median pseudotime values per cluster, used in figures depicting the thymic and BM datasets.

### Differential gene expression (DGE) analysis

For all datasets, DEGs between WT and Aire-deficient mice in each cluster were calculated using Seurat’s FindMarkers function. Only genes expressed in at least 10% of the cells in cluster (min.pct = 0.1) were evaluated, with a minimum difference in expression between the groups of log2fc 0.1. Differential expressions were tested using the Wilcoxon rank sum test with Bonferroni correction, and all genes satisfying the conditions log2fc ± 0.5 and adjusted *p* < 0.05 were regarded as DEGs.

GO analysis of DEGs in clusters with more than 10 DEGs was performed using the enrichGO function in *clusterProfiler* v.4.10.0 with Entrez IDs found with *AnnotationDbi* v.1.64.1^50^. We narrowed the search to ont = ‘BP’, performing the analysis using both (1) all DEGs together and (2) downregulated and upregulated genes separately, with nearly identical results. Enriched terms with less than two genes constituting the enrichment were filtered out.

### Thymic IFN signature and GO-based module scores

To quantify the effect of reduced IFN stimulation in late-late stage thymocyte populations, we designed a thymic IFN module score based on ISG DEGs with the largest downregulation in late-stage thymocytes in Aire-deficient mice. To identify these DEGs, gene counts from all cells in the subsetted late-stage thymocyte object were aggregated for each mouse, and genes with less than 10 counts across all mice were removed. Differential gene expression was performed with *DESeq2* v.1.42.0 using test: ‘Wald’ and fitType: ‘parametric’^51^. Results were then filtered, removing genes where either *p*.value and/or p.adj yielding NA, being genes with low counts detected by extreme count outlier detection in *DESeq2*. Out of 105 downregulated genes with log2fc ± 0.5 and adj.*p*.value < 0.05, we filtered to retain DEGs constituting the ‘response to virus’ GO term (GO:0009615) and selected 15 genes with the lowest log2fc values; *Oasl2, Ifit3, Ifi27l2a, Rtp4, Ifit1, Usp18, Isg15, Irf7, Ifi213, Ifi214, Bst2, Ddx60, Oas3, Isg20 and Ifi206*. We then used the AddModuleScore function in *Seurat* with these 15 genes as input to obtain a thymic IFN module score for each cell. The scores were compared for cell clusters across WT and Aire^C313X−/−^ using the Wilcoxon rank sum test with Bonferroni correction.

For mTEC, LN and BM datasets, we additionally employed GO-based scores for type I IFN, type II IFN and antigen processing and presentation, based on all genes within GO terms of interest (GO: 0034340, GO: 0034341 and GO:0019882), used as input genes for the AddModuleScore function in *Seurat*. The scores were compared in cell populations for Aire^C313X−/−^ and WT mice using the Wilcoxon rank sum test with Bonferroni correction.

### TCR-seq analysis

Filtered_contig_annotation.csv TCR-seq output files from CellRanger were loaded into RStudio and manually filtered to only retain transcripts from clones that have passed QC and that were annotated as thymocytes in the GEX dataset. Clones for which (1) no TCR transcripts were recovered, (2) only TCRα receptor transcript was recovered (missing TCRβ) or (3) clones with more than one transcript of either TCRα and/or TCRβ chain were annotated as ‘no_valid_TCR_info’ and excluded from further TCR analysis. Clones that we deemed biologically relevant/interpretable were therefore the ones containing either a single TCRβ transcript or a pair-chained receptor.

We defined a clonotype based on shared V and J genes used, as well as complementary determining region (CDR3) nucleotide (nt) sequence, according to recommendations by EuroClonality NGS Working Group^52^. For clones where only the TCRβ transcript was present, the clonotype identifiers for TCRα were collectively assigned as ‘NA’ and retained for unique clone frequency and diversity estimation. Unique clone frequency is the abundance of clonotypes in a sample consisting of one clone. Diversity was estimated using Shannon diversity index (H)

$$H=-{\sum }_{i=1}^{s}{p}_{{{{\rm{i}}}}} \, {{{\mathrm{ln}}}} \, {p}_{{{{\rm{i}}}}} \, {where} \, {p}_{{{{\rm{i}}}}}=\frac{{n}_{{{{\rm{i}}}}}}{N}$$

*s* = number of unique clonotypes (species)

${n}_{{{{\rm{i}}}}}\,$= number of clones in clonotype

*N* = total number of clones in sample

V and J gene usage was calculated for all clones with valid TCR, expressed as proportion of cells using a given gene out of all clones expressing TCRα for (*Trav* and *Traj*) or TCRβ (*Trbv* and *Trbj*).

We used *Immunarch* v.0.9.0 for clonal similarity/overlap estimation across samples. We only considered clones with paired-chain receptor data in this analysis, by loading the manually pre-filtered filtered_contig_annotation.csv files using repLoad() with .mode = ‘paired’. We then used repOverlap() with .method = ‘morisita’ and either .col = ‘nt+v + j’ or col. = ‘aa’ to estimate overlap based on either shared clonotypes or the CDR3 amino acid sequences in repertoires.

### Quantitative real-time PCR (qPCR) verification of differentially expressed genes

Cryopreserved thymic, BM and LN immune cells were thawed, washed twice (300 g × 5 min) in PBS and lysed on an RNA QIAshredder column (QIAGEN GmbH, Hilden, Germany, cat. 79656) with 350 μL RLT lysis buffer from RNeasy Mini Kit (QIAGEN GmbH, Hilden, Germany, cat. 74106). RNA isolation was performed using the RNeasy Mini Kit, including DNA digestion with RNAse-Free DNase Set (QIAGEN GmbH, Hilden, Germany, cat. 79256) as specified by the manufacturer’s protocols. Five hundred nanograms of isolated RNA was used as input for cDNA synthesis using Superscript IV VILO Kit with EZ DNase enzyme (Thermo Fisher Scientific, Waltham, MA, USA, cat. 11766050), including a gDNA digestion step, according to the manufacturer’s protocol. Synthesised cDNA was diluted 1:100 in nuclease-free water to be used for qPCR reactions.

Expression of *Ifi27l2a*, *Isg15*, *Rtp4*, *Irf7*, *Usp18*, *Stat1, RTP4* and *Ly6a* was measured using PowerTrack SYBR Green Master Mix (Thermo Fisher Scientific, Waltham, MA, USA, cat. A46109) according to the manufacturer’s protocol. Gene-specific primers (Merck KGaA, Darmstadt, Germany) are listed in Table 1, where 5% v/v was used per reaction. All samples were run in technical triplicates for 40 cycles in standard cycling mode, analysed using the QuantStudio5 system (Thermo Fisher Scientific, Waltham, MA, USA, cat. A28140). Mean expression of housekeeping genes; *Actb*, *Gadh* and *Hprt* was used for within-sample normalisation. Relative gene expression was calculated using the 2^−∆∆Ct^ method, and the mean of Ct values for WT samples was used as the calibrator for fold changes in expression across samples. Statistics were calculated in Prism version 9 (Graph Pad Software, Inc., San Diego, CA, USA) using the Wilcoxon rank sum test.

**Table 1:** Primers used for qPCR

Gene	Forward (5′ –> 3′)	Reverse (5′ –> 3′)
Actb	GGCTGTATTCCCCTCCATCG	CCAGTTGGTAACAATGCCATGT
Gadph	AGGTCGGTGTGAACGGATTTG	TGTAGACCATGTAGTTGAGGTCA
Hprt	TCAGTCAACGGGGGACATAAA	GGGGCTGTACTGCTTAACCAG
Ifi27l2a	GAACACTGTTTGGCTCTG	CTGCTGATTGGAGTGTGGCTAC
Irf7	GAGACTGGCTATTGGGGGAG	GACCGAAATGCTTCCAGGG
Isg15	GGTGTCCGTGACTAACTCCAT	TGGAAAGGGTAAGACCGTCCT
Ly6a	AGGAGGCAGCAGTTATTGTGG	CGTTGACCTTAGTACCCAGGA
Rtp4	TGGGAGCAGACATTTCAAGAAC	ACCTGAGCAGAGGTCCAACTT
Stat1	TCACAGTGGTTCGAGCTTCAG	GCAAACGAGACATCATAGGCA
Usp18	TTGGGCTCCTGAGGAAACC	CGATGTTGTGTAAACCAACCAGA

### Statistics and reproducibility

For mouse experiments conducted in this study, four biological replicates (of similar age and same sex across groups) were used. For qPCR experiments 3–5 biological replicates were used per genotype, and each measurement was conducted in technical triplicates. For gene expression analyses with Seurat’s tools to find conserved marker- and DEGs, Wilcoxon rank sum test with Bonferroni correction was used. Other comparisons (cluster abundance, TCR gene usage, repertoire diversity metrics, gene expression analyses by qPCR and IFN module score differences across genotypes) were performed using the Wilcoxon rank sum test with Benjamini–Hochberg correction. For GO term enrichment, an over-representation test, which is a version of the Fisher exact test with Benjamini–Hochberg correction was used. The number of cells from each sample in clusters, descriptive statistics and results of statistical tests can be found in supplementary data files.

### Reporting summary

Further information on research design is available in the Nature Portfolio Reporting Summary linked to this article.

## Supplementary information

Supplementary Information

Description of Additional Supplementary Materials

Supplementary Data 1

Supplementary Data 2

Supplementary Data 3

Supplementary Data 4

Reporting Summary

## Data Availability

Sequencing data are available through the ArrayExpress database (http://www.ebi.ac.uk/arrayexpress), accession number E-MTAB-14774. All other data are available upon request.

## References

[1] Anderson, M. S. et al. Projection of an immunological self shadow within the thymus by the Aire protein. *Science* **298**, 1395–1401 (2002). 12376594 10.1126/science.1075958

[2] Liston, A., Lesage, S., Wilson, J., Peltonen, L. & Goodnow, C. C. Aire regulates negative selection of organ-specific T cells. *Nat. Immunol.* **4**, 350–354 (2003). 12612579 10.1038/ni906

[3] Liston, A. et al. Gene dosage–limiting role of Aire in thymic expression, clonal deletion, and organ-specific autoimmunity. *J. Exp. Med.* **200**, 1015–1026 (2004). 15492124 10.1084/jem.20040581 PMC2211852

[4] Anderson, M. S. et al. The cellular mechanism of Aire control of T cell tolerance. *Immunity* **23**, 227–239 (2005). 16111640 10.1016/j.immuni.2005.07.005

[5] Aaltonen, J. et al. An autoimmune disease, APECED, caused by mutations in a novel gene featuring two PHD-type zinc-finger domains. *Nat. Genet.* **17**, 399–403 (1997). 9398840 10.1038/ng1297-399

[6] Nagamine, K. et al. Positional cloning of the APECED gene. *Nat. Genet.* **17**, 393–398 (1997). 9398839 10.1038/ng1297-393

[7] Laan, M. et al. Autoimmune regulator deficiency results in decreased expression of CCR4 and CCR7 ligands and in delayed migration of CD4+ thymocytes1. *J. Immunol.* **183**, 7682–7691 (2009). 19923453 10.4049/jimmunol.0804133 PMC2795747

[8] Lei, Y. et al. Aire-dependent production of XCL1 mediates medullary accumulation of thymic dendritic cells and contributes to regulatory T cell development. *J. Exp. Med.* **208**, 383–394 (2011). 21300913 10.1084/jem.20102327 PMC3039864

[9] Ashby, K. M. et al. Sterile production of interferons in the thymus affects T cell repertoire selection. *Sci. Immunol.* **9**, eadp1139 (2024). 39058762 10.1126/sciimmunol.adp1139 PMC12052003

[10] Benhammadi, M. et al. IFN-λ enhances constitutive expression of MHC class I molecules on thymic epithelial cells. *J. Immunol.* **205**, 1268–1280 (2020). 32690660 10.4049/jimmunol.2000225

[11] Cowan, J. E. et al. Aire controls the recirculation of murine Foxp3+ regulatory T-cells back to the thymus. *Eur. J. Immunol.* **48**, 844–854 (2018). 29285761 10.1002/eji.201747375 PMC6001551

[12] Lienenklaus, S. et al. Novel reporter mouse reveals constitutive and inflammatory expression of IFN-β in vivo1. *J. Immunol.* **183**, 3229–3236 (2009). 19667093 10.4049/jimmunol.0804277

[13] Ashby, K. M. & Hogquist, K. A. A guide to thymic selection of T cells. *Nat. Rev. Immunol.* **24**, 103–117 (2024). 37464188 10.1038/s41577-023-00911-8

[14] Breed, E. R., Lee, S. T. & Hogquist, K. A. Directing T cell fate: how thymic antigen presenting cells coordinate thymocyte selection. *Semin. Cell Dev. Biol.* **84**, 2–10 (2018). 28800929 10.1016/j.semcdb.2017.07.045 PMC5807247

[15] Stritesky, G. L., Jameson, S. C. & Hogquist, K. A. Selection of self-reactive T cells in the thymus. *Annu. Rev. Immunol.* **30**, 95–114 (2012). 22149933 10.1146/annurev-immunol-020711-075035 PMC3518413

[16] Yang, S., Fujikado, N., Kolodin, D., Benoist, C. & Mathis, D. Regulatory T cells generated early in life play a distinct role in maintaining self-tolerance. *Science* **348**, 589–594 (2015). 25791085 10.1126/science.aaa7017 PMC4710357

[17] Dong, J. et al. Homeostatic properties and phenotypic maturation of murine CD4+ pre-thymic emigrants in the thymus. *PLoS ONE* **8**, e56378–e56378 (2013). 23409179 10.1371/journal.pone.0056378 PMC3569422

[18] Li, J. et al. Developmental pathway of CD4+CD8- medullary thymocytes during mouse ontogeny and its defect in Aire -/- mice. *Proc. Natl. Acad. Sci. USA* **104**, 18175–18180 (2007). 10.1073/pnas.0708884104 PMC2084316 17984055

[19] Mouri, Y. et al. Mode of tolerance induction and requirement for Aire are governed by the cell types that express self-antigen and those that present antigen. *J. Immunol.* **199**, 3959–3971 (2017). 29101311 10.4049/jimmunol.1700892

[20] Oftedal, B. E. et al. A partial form of AIRE deficiency underlies a mild form of autoimmune polyendocrine syndrome type 1. *J. Clin. Investig.* **133**, e169704 (2023). 10.1172/JCI169704 PMC10617782 37909333

[21] Jin, R. et al. Critical role of SP thymocyte motility in regulation of thymic output in neonatal Aire ^-/-^ mice. *Oncotarget* **8**, 83 (2016). 10.18632/oncotarget.13909 PMC5352200 27965471

[22] Gardner, J. M. et al. Deletional tolerance mediated by extrathymic Aire-expressing cells. *Science* **321**, 843–847 (2008). 18687966 10.1126/science.1159407 PMC2532844

[23] Eldershaw, S. A., Sansom, D. M. & Narendran, P. Expression and function of the autoimmune regulator (Aire) gene in non-thymic tissue. *Clin. Exp. Immunol.* **163**, 296–308 (2011). 21303359 10.1111/j.1365-2249.2010.04316.x PMC3048612

[24] Gardner, J. M. et al. Extrathymic Aire-expressing cells are a distinct bone marrow-derived population that induce functional inactivation of CD4⁺ T cells. *Immunity* **39**, 560–572 (2013). 23993652 10.1016/j.immuni.2013.08.005 PMC3804105

[25] Cohen, J. N. et al. Lymph node–resident lymphatic endothelial cells mediate peripheral tolerance via Aire-independent direct antigen presentation. *J. Exp. Med.* **207**, 681–688 (2010). 20308365 10.1084/jem.20092465 PMC2856027

[26] Trapnell, C. et al. The dynamics and regulators of cell fate decisions are revealed by pseudotemporal ordering of single cells. *Nat. Biotechnol.* **32**, 381–386 (2014). 24658644 10.1038/nbt.2859 PMC4122333

[27] Nishijima, H. et al. Aire controls heterogeneity of medullary thymic epithelial cells for the expression of self-antigens. *J. Immunol.* **208**, 303–320 (2022). 34930780 10.4049/jimmunol.2100692

[28] Michelson, D. A., Hase, K., Kaisho, T., Benoist, C. & Mathis, D. Thymic epithelial cells co-opt lineage-defining transcription factors to eliminate autoreactive T cells. *Cell* **185**, 2542–2558 (2022). 35714609 10.1016/j.cell.2022.05.018 PMC9469465

[29] Goldfarb, Y. et al. Mechanistic dissection of dominant AIRE mutations in mouse models reveals AIRE autoregulation. *J. Exp. Med.* **218**, e20201076 (2021). 10.1084/jem.20201076 PMC8421262 34477806

[30] St-Pierre, C., Trofimov, A., Brochu, S., Lemieux, S. & Perreault, C. Differential features of AIRE-induced and AIRE-independent promiscuous gene expression in thymic epithelial cells. *J. Immunol.* **195**, 498–506 (2015). 26034170 10.4049/jimmunol.1500558

[31] Xing, Y., Wang, X., Jameson, S. C. & Hogquist, K. A. Late stages of T cell maturation in the thymus involve NF-κB and tonic type I interferon signaling. *Nat. Immunol.* **17**, 565–573 (2016). 27043411 10.1038/ni.3419 PMC4837029

[32] Lee, C.-K., Gimeno, R. & Levy, D. E. Differential regulation of constitutive major histocompatibility complex class I expression in T and B lymphocytes. *J. Exp. Med.* **190**, 1451–1464 (1999). 10562320 10.1084/jem.190.10.1451 PMC2195695

[33] James, K. D., Jenkinson, W. E. & Anderson, G. T-cell egress from the thymus: should I stay or should I go? *J. Leukoc. Biol.* **104**, 275–284 (2018). 29485734 10.1002/JLB.1MR1217-496R PMC6174998

[34] Mohammed, A. et al. Distinct type I and II interferon responses direct cortical and medullary thymic epithelial cell development. *Sci. Immunol.* **10**, eado4720 (2025). 40315299 10.1126/sciimmunol.ado4720

[35] Martinez, R. J. et al. Type III interferon drives thymic B cell activation and regulatory T cell generation. *Proc. Natl. Acad. Sci. USA* **120**, e2220120120 (2023). 36802427 10.1073/pnas.2220120120 PMC9992806

[36] You, Y. et al. Direct presentation of inflammation-associated self-antigens by thymic innate-like T cells induces elimination of autoreactive CD8+ thymocytes. *Nat. Immunol.* **25**, 1367–1382 (2024). 38992254 10.1038/s41590-024-01899-6 PMC11291280

[37] Wang, J. et al. Hassall’s corpuscles with cellular-senescence features maintain IFNalpha production through neutrophils and pDC activation in the thymus. *Int Immunol.* **31**, 127–139 (2019). 30534943 10.1093/intimm/dxy073 PMC9271218

[38] Stoljar, A. et al. Impaired Aire-dependent IFN signaling in the thymus precedes the protective autoantibodies to IFNalpha. *J. Exp. Med.* **222**, e20241403 (2025). 10.1084/jem.20241403 PMC12042843 40304722

[39] Kärner, J. et al. Anti-cytokine autoantibodies suggest pathogenetic links with autoimmune regulator deficiency in humans and mice. *Clin. Exp. Immunol.* **171**, 263–272 (2013). 23379432 10.1111/cei.12024 PMC3569533

[40] Kisand, K. et al. Interferon autoantibodies associated with AIRE deficiency decrease the expression of IFN-stimulated genes. *Blood* **112**, 2657–2666 (2008). 18606876 10.1182/blood-2008-03-144634 PMC2577576

[41] Meager, A. et al. Anti-interferon autoantibodies in autoimmune polyendocrinopathy syndrome type 1. *PLoS Med.* **3**, e289–e289 (2006). 16784312 10.1371/journal.pmed.0030289 PMC1475653

[42] Oftedal, B. E., Sjøgren, T. & Wolff, A. S. B. Interferon autoantibodies as signals of a sick thymus. *Front. Immunol.* **15**, 1327784 (2024). 38455040 10.3389/fimmu.2024.1327784 PMC10917889

[43] Hao, Y. et al. Integrated analysis of multimodal single-cell data. *Cell* **184**, 3573–3587 (2021). 34062119 10.1016/j.cell.2021.04.048 PMC8238499

[44] Li, Y. et al. Development of double-positive thymocytes at single-cell resolution. *Genome Med.* **13**, 49 (2021). 33771202 10.1186/s13073-021-00861-7 PMC8004397

[45] Steier, Z. et al. Single-cell multiomic analysis of thymocyte development reveals drivers of CD4+ T cell and CD8+ T cell lineage commitment. *Nat. Immunol.* **24**, 1579–1590 (2023). 37580604 10.1038/s41590-023-01584-0 PMC10457207

[46] Park, J.-E. et al. A cell atlas of human thymic development defines T cell repertoire formation. *Science* **367**, eaay3224 (2020). 32079746 10.1126/science.aay3224 PMC7611066

[47] McGinnis, C. S., Murrow, L. M. & Gartner, Z. J. DoubletFinder: doublet detection in single-cell RNA sequencing data using artificial nearest neighbors. *Cell Syst.* **8**, 329–337 (2019). 30954475 10.1016/j.cels.2019.03.003 PMC6853612

[48] Stuart, T. et al. Comprehensive integration of single-cell data. *Cell* **177**, 1888–1902 (2019). 31178118 10.1016/j.cell.2019.05.031 PMC6687398

[49] McInnes, L., Healy, J. & Melville, J. Umap: uniform manifold approximation and projection for dimension reduction. Preprint at 10.48550/arXiv.1802.03426 (2018).

[50] Wu, T. et al. ClusterProfiler 4.0: a universal enrichment tool for interpreting omics data. *Innovation* **2**, 100141 (2021). 10.1016/j.xinn.2021.100141 PMC8454663 34557778

[51] Love, M. I., Huber, W. & Anders, S. Moderated estimation of fold change and dispersion for RNA-seq data with DESeq2. *Genome Biol.* **15**, 550 (2014). 25516281 10.1186/s13059-014-0550-8 PMC4302049

[52] Sofou, E. et al. Clonotype definitions for immunogenetic studies: proposals from the EuroClonality NGS Working Group. *Leukemia* **37**, 1750–1752 (2023). 37391484 10.1038/s41375-023-01952-7 PMC10400411

