# Supplementary material for: Aire-dependent interferon signalling shapes thymocyte maturation and central tolerance in mice
Source: Commun Biol. 2025 Dec 9;9:53. doi: 10.1038/s42003-025-09317-9 (PMC12795847; doi:10.1038/s42003-025-09317-9)
Supplement: Supplementary file 1 — Supplementary Information [file 42003_2025_9317_MOESM1_ESM.pdf]

Supplemental figures for

# Aire-dependent interferon signalling shapes thymocyte maturation and central tolerance in mice

Adrianna Jebrzycka[1], Lars Breivik[1,2], David Dolan[3], Yael Goldfarb[4], Jakub Abramson[4], Anette S. B Wolff[1,2,5], Eystein S. Husebye[1,2], Anagha M. Joshi[1,3], Bergithe E. Oftedal[1,2]

1. Department of Clinical Science, University of Bergen, Bergen, Norway
2. Department of Medicine, Haukeland University hospital, Bergen, Norway
3. Department of Informatics, Computational Biology Unit, University of Bergen, Norway
4. Department of Immunology and Regenerative Biology, Weizmann Institute of Science, Rehovot, Israel
5. Health Research Sogn og Fjordane, Førde Hospital Trust, Førde, Norway

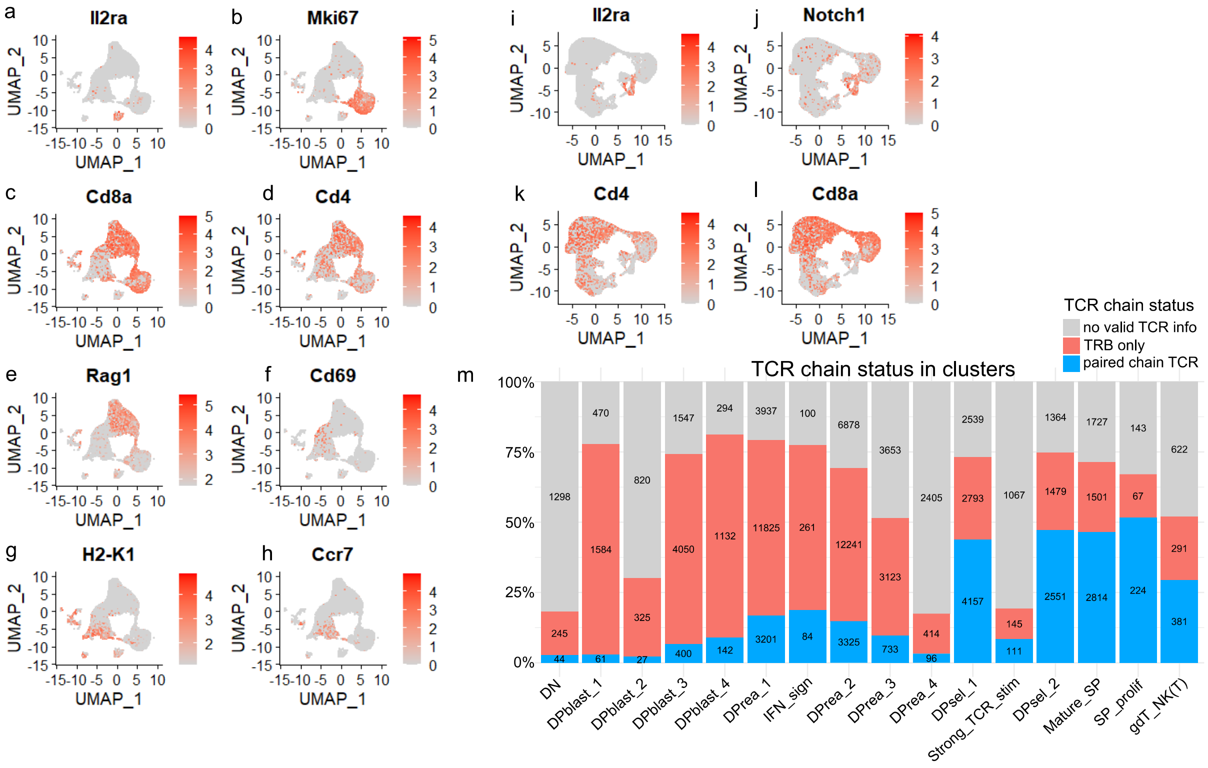


**Supplementary figure 1. Markers of thymocyte development and overview over T cell receptor (TCR) chain status in TCR-seq data.** a-h) UMAP plots depicting the expression of *Il2ra*, *Mki67*, *Cd8a*, *Cd4*, *Rag1*, *Cd69*, *H2-K1* and *Ccr7* in the whole thymus immune cell object. I-l) Expression of *Il2ra*, *Notch1*, *Cd4* and *Cd8* in the subsetted thymocyte object used for developmental trajectory analysis. m) Proportion of thymocyte clones with red: only TCRβ chain, blue: paired-chain TCR or grey: clones with absent TCR transcript information or expressing more than one α or β chain in clusters ordered by developmental stage. Numbers in bars indicate absolute number of clones in category.

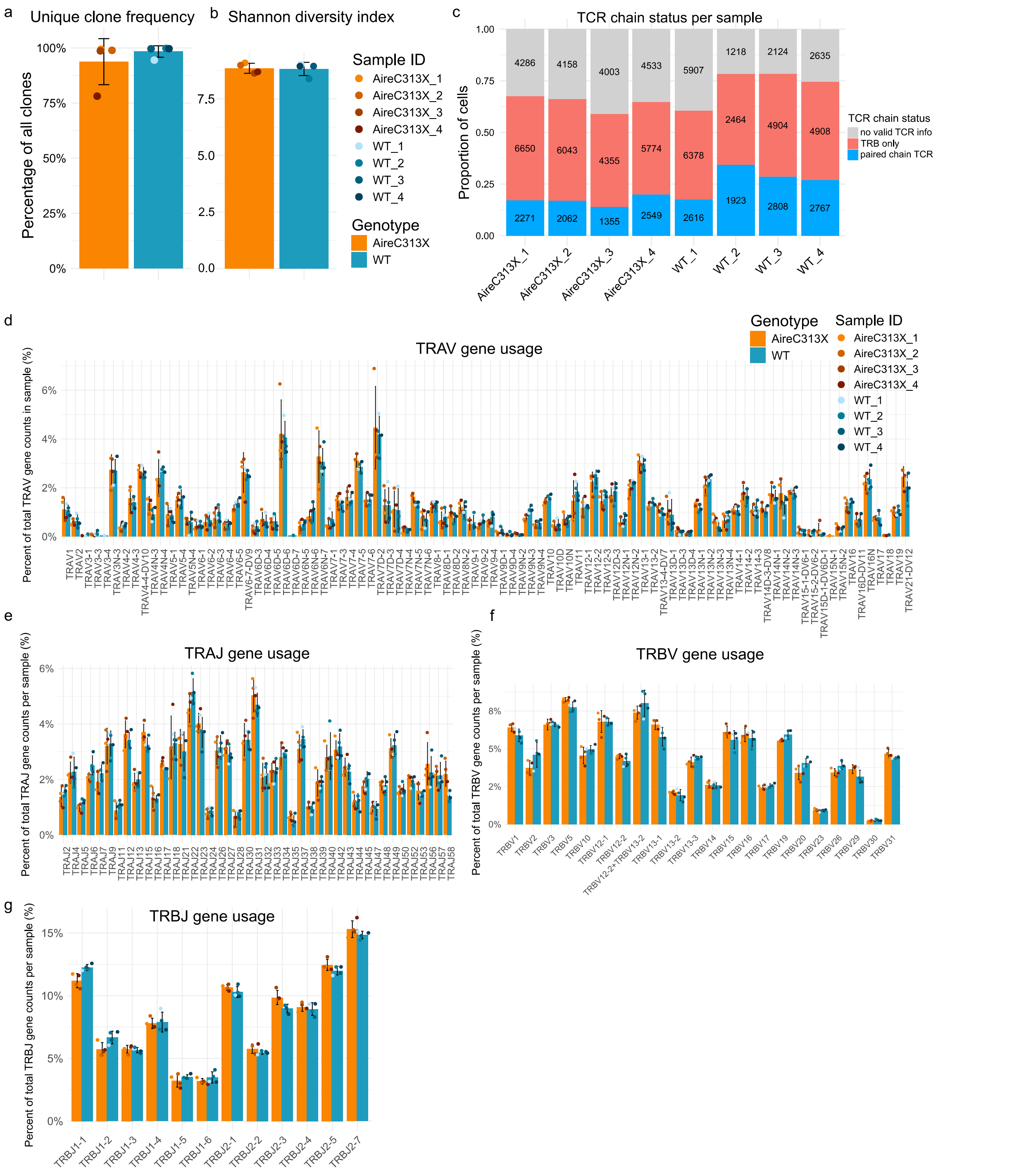


**Supplementary figure 2. TCR repertoire analysis of Aire$^{C313X-/-}$ and WT thymocytes.** a) Abundance of clonotypes consisting of one clone (unique clone). Clonotype defined as clone expressing the same V+J gene and CDR3 nucleotide sequence from TRB and TRA (if TRA transcript was present, otherwise annotated “NA”, Wilcoxon rank sum test, ns). b) Clonal diversity in Aire$^{C313X-/-}$ and WT mice estimated using Shannon diversity index (Wilcoxon rank sum test, ns). c) Proportion of clones per sample with red: only TCRβ chain, blue: paired-chain TCR or grey: clones with absent TCR transcript info or expressing more than one transcript of α or β chain. Numbers in bars indicate absolute clone numbers in category. Relative frequency of clones per sample using the specified d) *Trav* and e) *Traj* f) *Trbv* and g) *Trbj* gene compared across Aire$^{C313X-/-}$ and WT mice (Wilcoxon rank sum test with Benjamini Hochberg correction, ns). In a,b,d,e,f; error bars in bar plots indicate standard deviation.

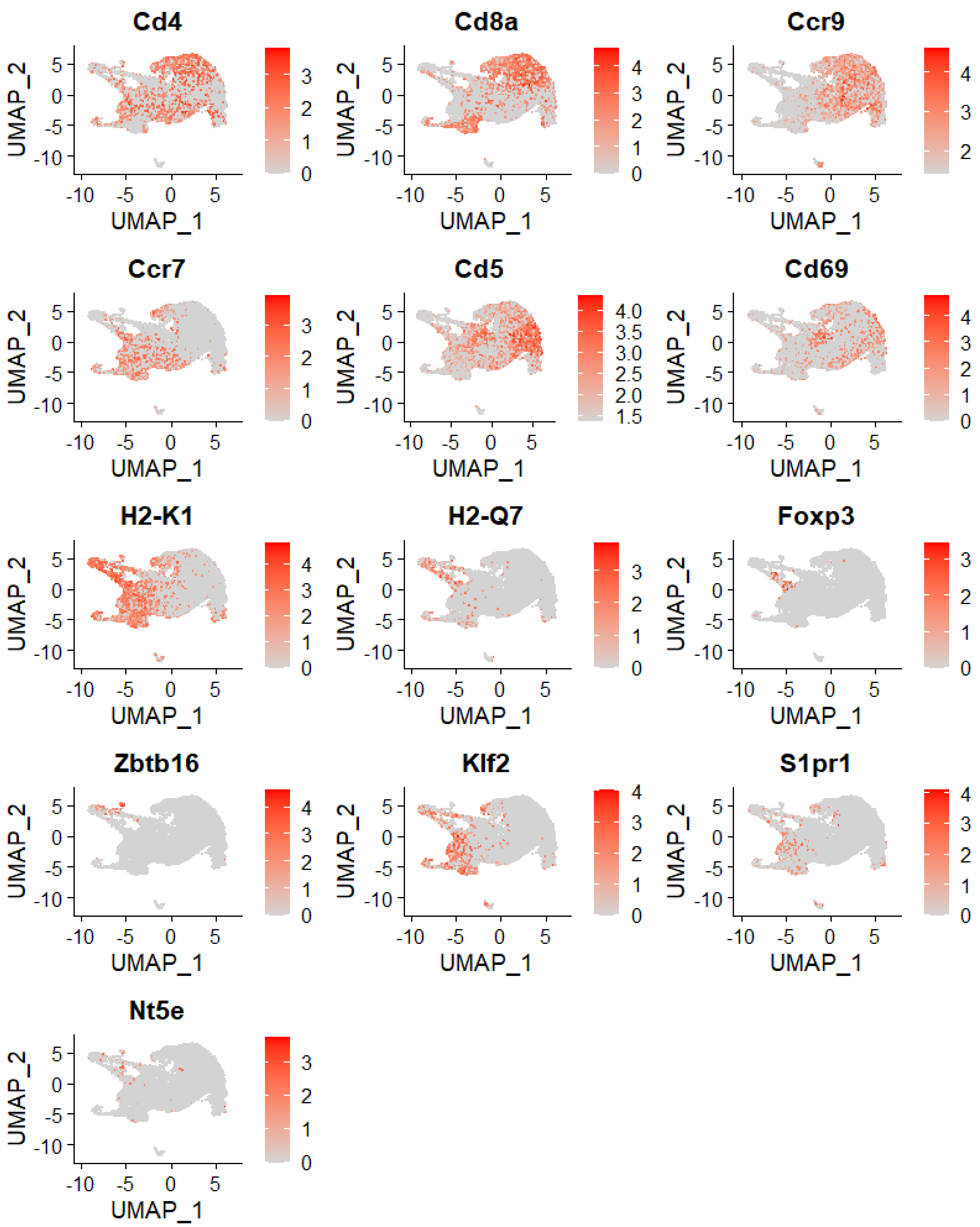


**Supplementary figure 3. Markers of late-stage thymocyte development in re-clustered late-stage thymocytes of Aire-deficient (Aire$^{C313X-/-}$) and wild-type (WT) mice.** a) Late-stage thymocytes consisting of cells annotated as DPsel 1, DPsel 2, Strong TCR stim, Mature SP, SP prolif and gdT/NK in the main thymus object (main fig.1a) were subsetted and re-clustered yielding 14 higher-resolution clusters. Expression of markers used to identify populations of late-stage and non-conventional thymocytes: *Cd4*, *Cd8a*, *Ccr9*, *Ccr7*, *Cd5*, *Cd69*, *H2-K1*, *H2-Q7* (Qa2), *Foxp3*, *Zbtb16* and thymic egress markers: *Klf2, S1pr1* and *Nt5e* (encoding Cd73).

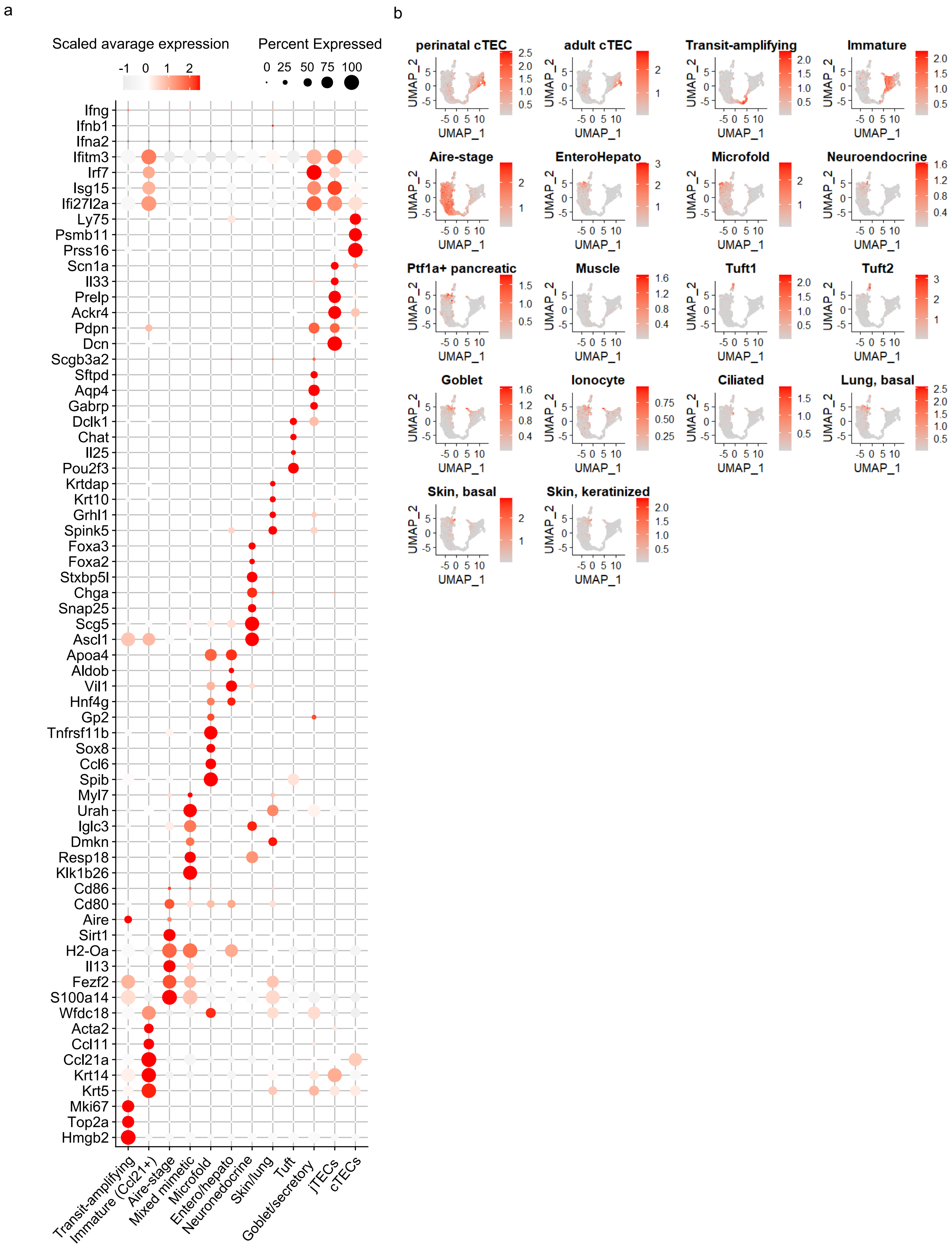


**Supplementary figure 4. Conserved marker genes for TEC populations from Aire knockout (AireKO) and wild-type (WT) mice (n = 2 per genotype, GSE155331).** a) Dotplot of conserved marker genes for the 12 TEC populations. The size of dot represents the percentage of cells in clusters expressing a gene, while the color represents the scaled average expression level. b) Gene set module scores of TEC and mimetic cell populations based on conserved gene lists from Michelson *et.al* [1]

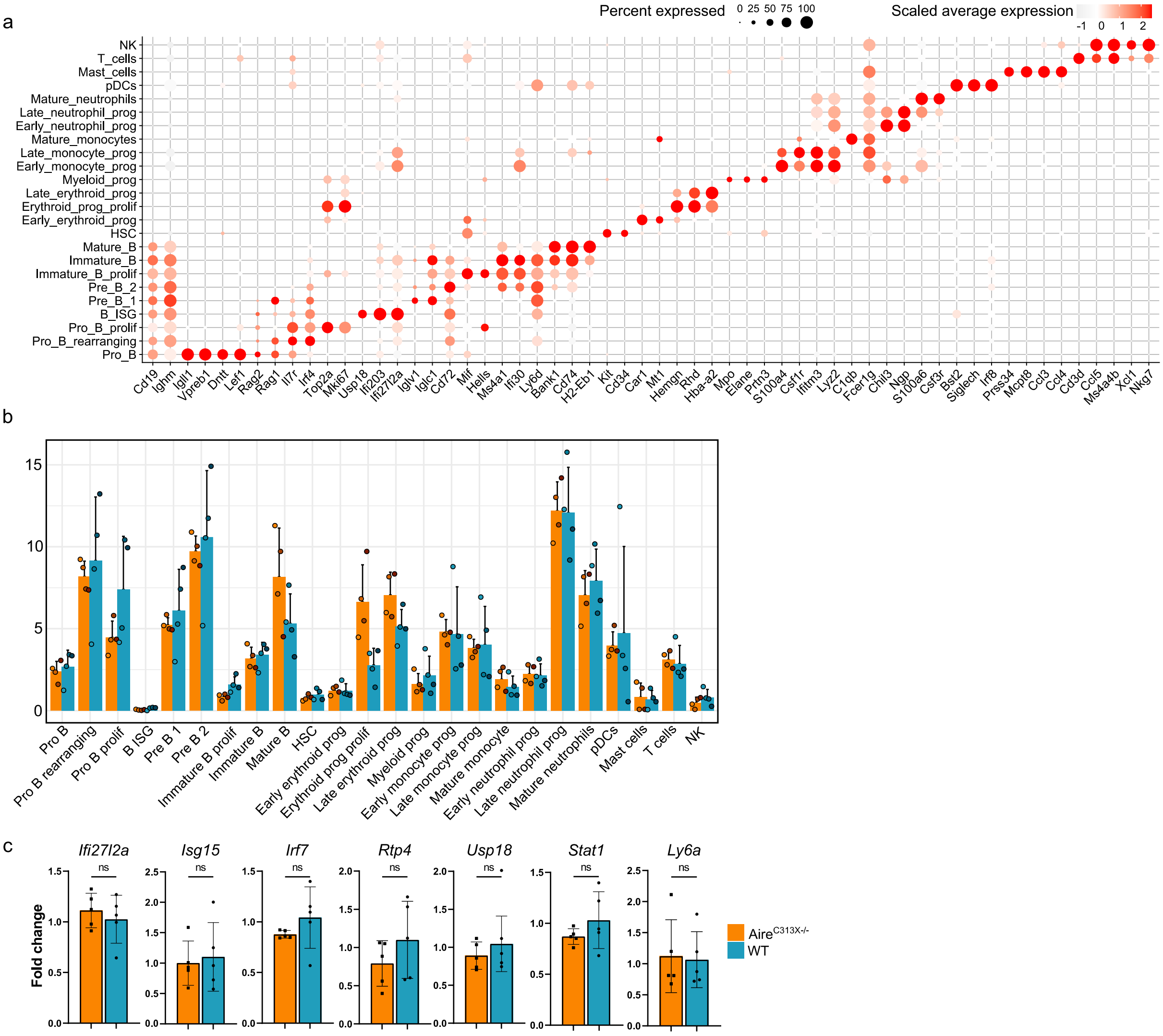

**Supplementary figure 5. Conserved marker genes, cell type abundances and interferon-stimulated gene (ISG) expression in bone marrow (BM) immune cells from Aire-deficient (Aire$^{C313X-/-}$) and wild-type (WT) mice.** Dotplots of conserved marker genes for BM immune cell populations. The size of dot represents the percentage of cells in clusters expressing a gene, while the color represents the scaled average expression level. b) Proportion of cells in clusters compared across Aire$^{C313X-/-}$ and WT (n=4 per genotype, Wilcoxon rank sum test with Benjamini-Hochberg correction, ns). c) Expression of ISGs *Ifi27l2a*, *Isg15*, *Irf7*, *Rtp4*, *Usp18*, *Stat1* and *Ly6a* quantified by qPCR in bulk immune cells from BM of Aire$^{C313X-/-}$ and WT (n=5 per genotype) mice (Wilcoxon rank sum test with Benjamini-Hochberg correction). Error bars in bar plots indicate standard deviation.

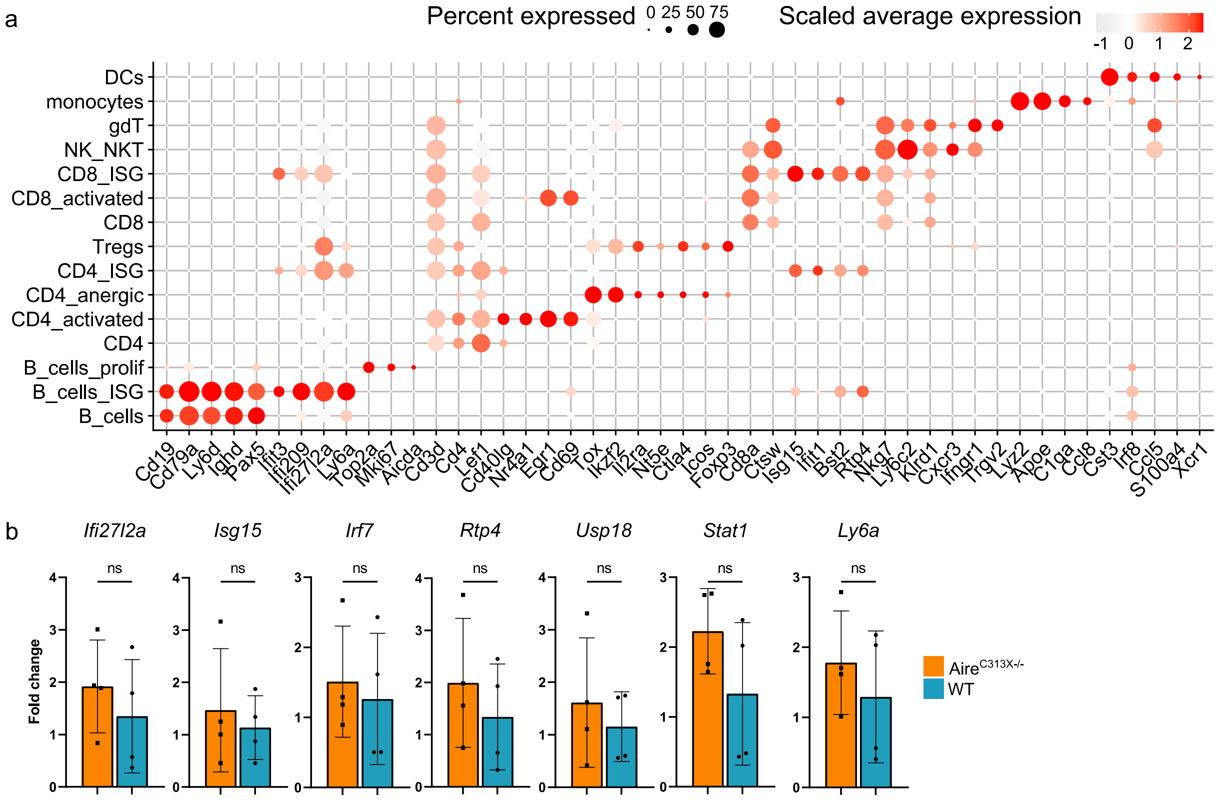


**Supplementary figure 6. Conserved marker gene and expression of interferon-stimulated gene (ISG) expression in lymph node (LN) immune cells from Aire-deficient (Aire$^{C313X-/-}$) and wild-type (WT) mice.** Dotplots of conserved marker genes for LN immune cell populations. The size of dot represents the percentage of cells in clusters expressing a gene, while the color represents the scaled average expression level. b) Expression of ISGs *Ifi27l2a*, *Isg15*, *Irf7*, *Rtp4*, *Usp18*, *Stat1* and *Ly6a* quantified by qPCR in bulk immune cells from LN of Aire$^{C313X-/-}$ and WT (n= 4 per group) mice (Wilcoxon rank sum test with Benjamini-Hochberg correction). Error bars in bar plots indicate standard deviation.

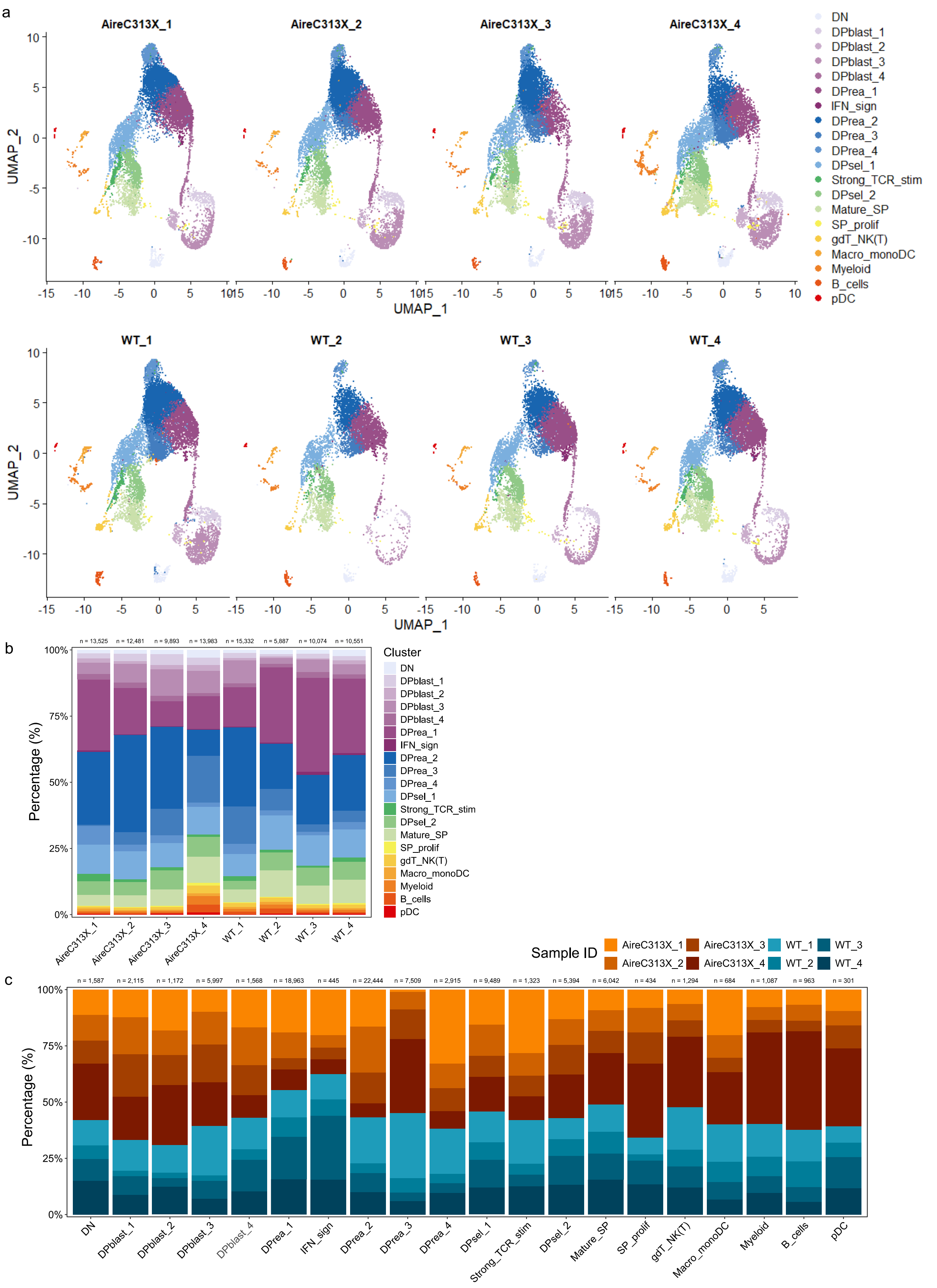


**Supplementary figure 7. Clusters of thymic immune cells after annotation and quality control from Aire-deficient (Aire$^{C313X-/-}$) and wild-type (WT) mice.** a) UMAP plot for each sample depicting clusters after quality control and cluster annotation. b) Proportion of cells in clusters shown for each mouse. c) Contribution of cells from each mouse to clusters in a shared UMAP space.

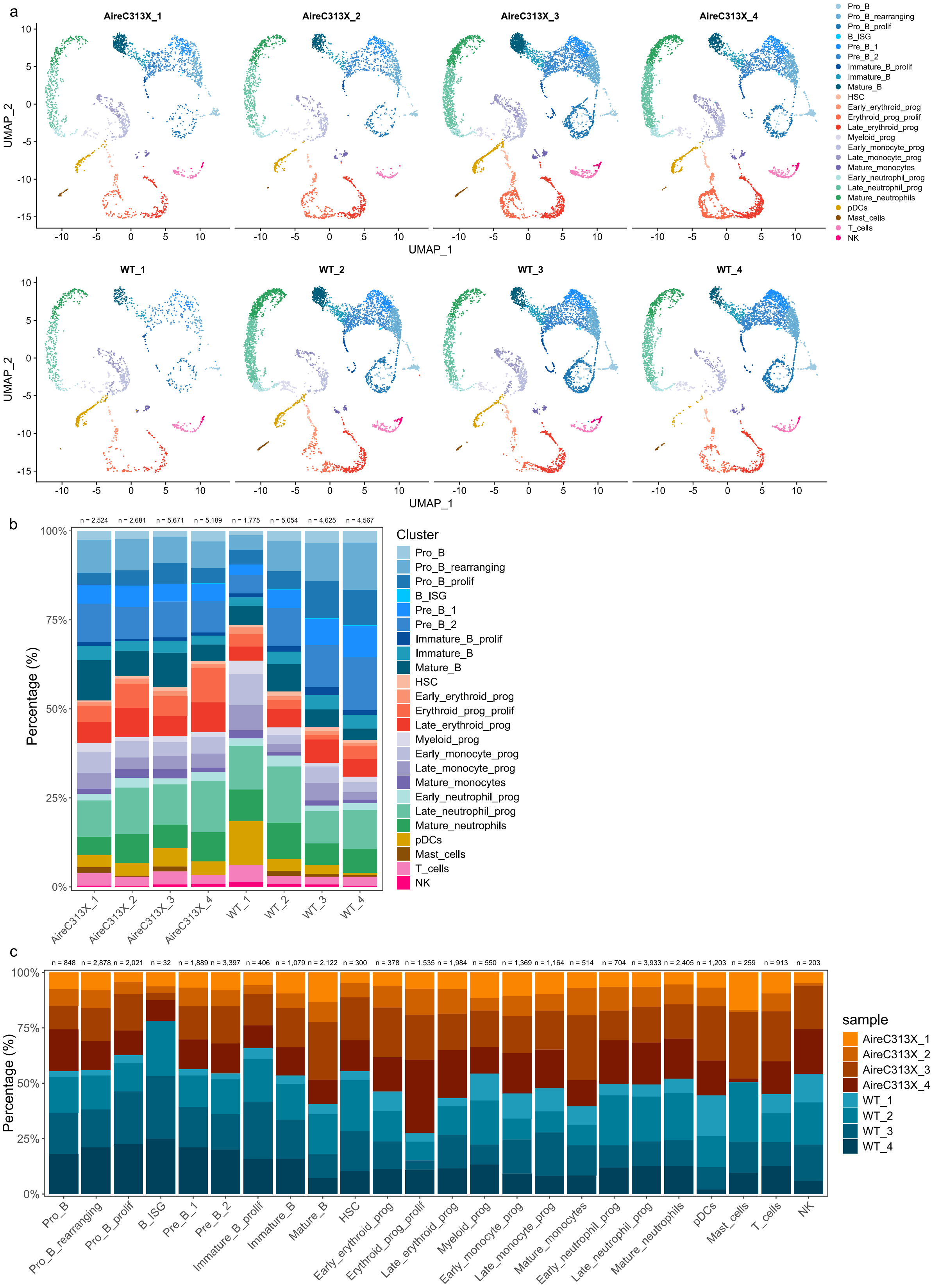


**Supplementary figure 8. Clusters of bone marrow immune cells after annotation and quality control from Aire-deficient (Aire$^{C313X-/-}$) and wild-type (WT) mice.** a) UMAP plot for each sample depicting clusters after quality control and cluster annotation. b) Proportion of cells in clusters shown for each mouse. c) Contribution of cells from each mouse to clusters in a shared UMAP space.

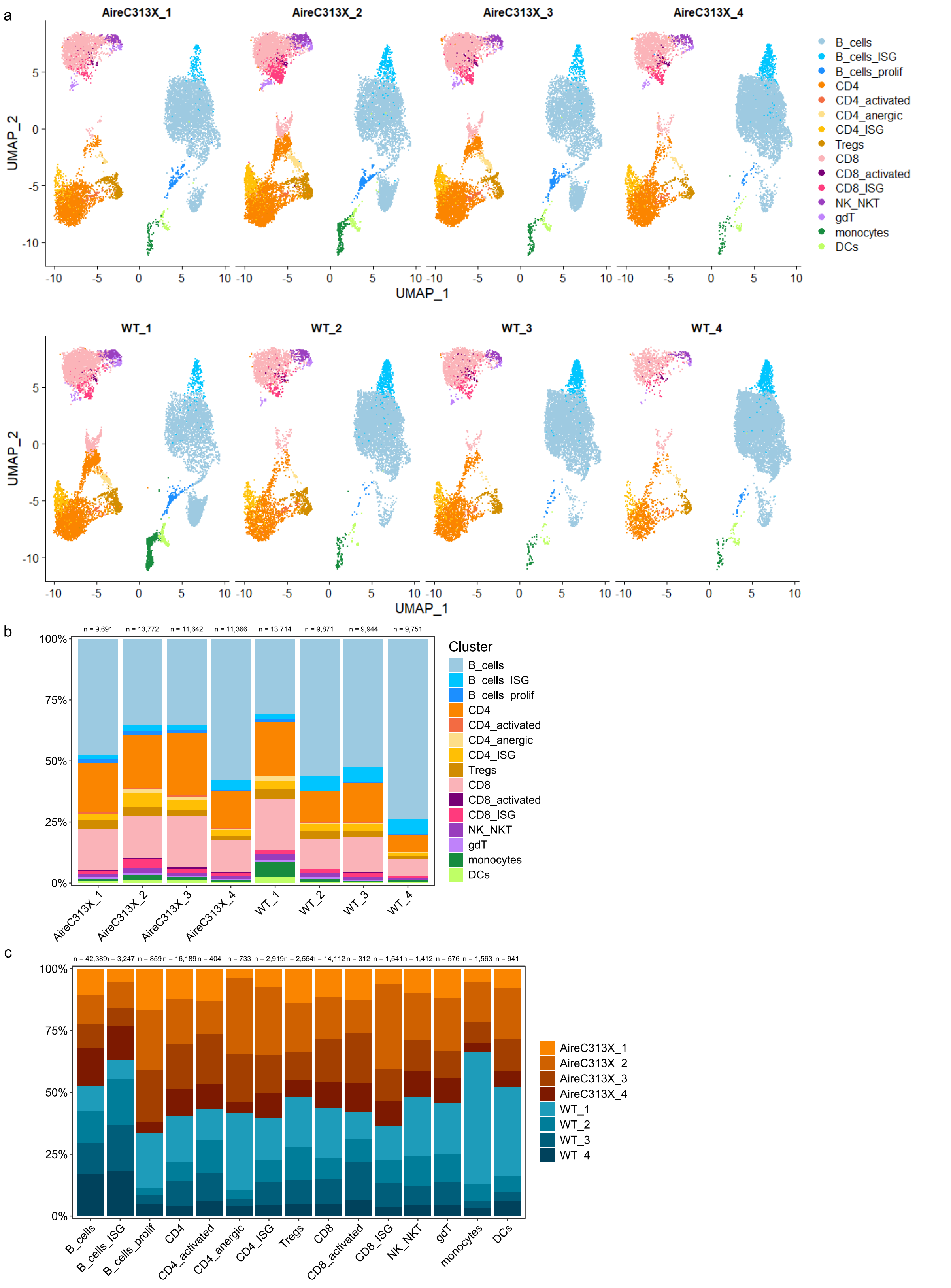


**Supplementary figure 9. Clusters of lymph node immune cells after annotation and quality control from Aire-deficient (Aire$^{C313X-/-}$) and wild-type (WT) mice.** a) UMAP plot for each sample depicting clusters after quality control and cluster annotation. b) Proportion of cells in clusters shown for each mouse. c) Contribution of cells from each mouse to clusters in a shared UMAP space.

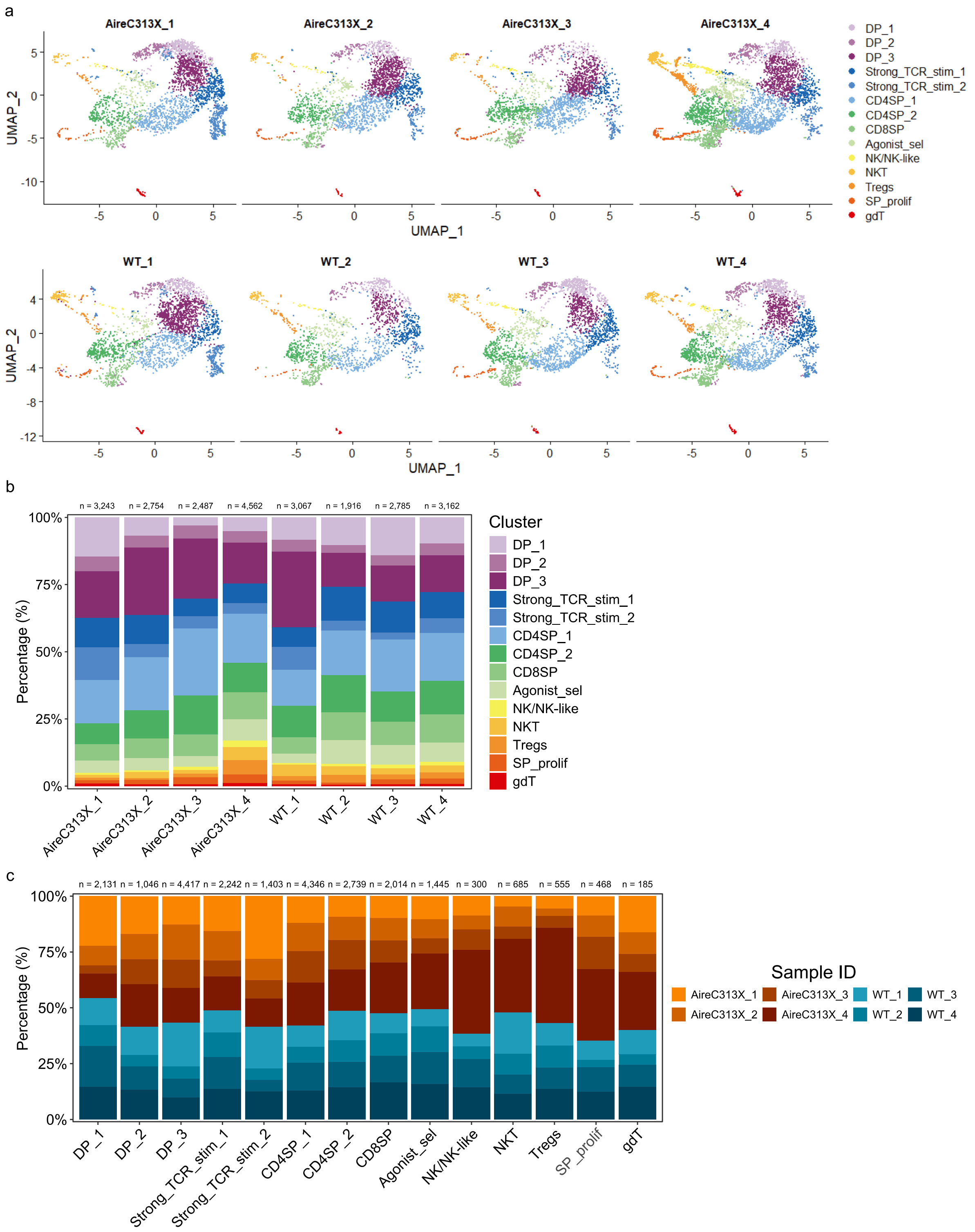


**Supplementary figure 10. Re-clustered late-stage thymocytes in Aire$^{C313X-/-}$ and WT mice.** a) UMAP plots of late-stage thymocyte clusters for each sample. b) Proportion of cells in clusters shown for each sample. c) Contribution of cells from each sample to clusters in a shared UMAP space

Reference:

1. Michelson DA, Hase K, Kaisho T, Benoist C, Mathis D. Thymic epithelial cells co-opt lineage-defining transcription factors to eliminate autoreactive T cells. *Cell* **185**, 2542-2558.e2518 (2022).
