# Supplementary material for: Aire-dependent interferon signalling shapes thymocyte maturation and central tolerance in mice
Source: Commun Biol. 2025 Dec 9;9:53. doi: 10.1038/s42003-025-09317-9 (PMC12795847; doi:10.1038/s42003-025-09317-9)
Supplement: Supplementary file 2 — Description of Additional Supplementary Materials [file 42003_2025_9317_MOESM2_ESM.pdf]

Description of Additional Supplementary Files

File name: Supplementary Data 1

Description: Source data for all single cell thymocyte analysis

File name: Supplementary Data 2

Description: Source data for all single cell bone marrow cell analysis

File name: Supplementary Data 3

Description: Source data for all single cell lymph node analysis

File name: Supplementary Data 4

Description: Source data for all single cell TEC analysis
